# Molecular gradients shape synaptic specificity of a visuomotor transformation

**DOI:** 10.1038/s41586-025-09037-4

**Published:** 2025-06-04

**Authors:** Mark Dombrovski, Yixin Zang, Giovanni Frighetto, Andrea Vaccari, HyoJong Jang, Parmis S. Mirshahidi, Fangming Xie, Piero Sanfilippo, Bryce W. Hina, Aadil Rehan, Roni H. Hussein, Pegah S. Mirshahidi, Catherine Lee, Aileen Morris, Mark A. Frye, Catherine R. von Reyn, Yerbol Z. Kurmangaliyev, Gwyneth M. Card, S. Lawrence Zipursky

**Affiliations:** 1 Department of Biological Chemistry, David Geffen School of Medicine, University of California Los Angeles, Los Angeles, CA USA; 2 Department of Neuroscience, Howard Hughes Medical Institute, The Mortimer B. Zuckerman Mind Brain Behavior Institute, Columbia University, New York, NY USA; 3 Department of Integrative Biology and Physiology, University of California Los Angeles, Los Angeles, CA USA; 4 Department of Computer Science, Middlebury College, Middlebury, VT USA; 5 School of Biomedical Engineering, Science and Health Systems, Drexel University, Philadelphia, PA USA; 6 Department of Biology, Brandeis University, Waltham, MA USA

**Keywords:** Neuronal development, Neural circuits, Molecular neuroscience

## Abstract

How does the brain convert visual input into specific motor actions^1,2^? In *Drosophila*, visual projection neurons (VPNs)^3,4^ perform this visuomotor transformation by converting retinal positional information into synapse number in the brain^5^. The molecular basis of this phenomenon remains unknown. We addressed this issue in LPLC2 (ref. ^6^), a VPN type that detects looming motion and preferentially drives escape behaviour to stimuli approaching from the dorsal visual field with progressively weaker responses ventrally. This correlates with a dorsoventral gradient of synaptic inputs into and outputs from LPLC2. Here we report that LPLC2 neurons sampling different regions of visual space exhibit graded expression of cell recognition molecules matching these synaptic gradients. Dpr13 shapes LPLC2 outputs by binding DIP-ε in premotor descending neurons mediating escape. Beat-VI shapes LPLC2 inputs by binding Side-II in upstream motion-detecting neurons. Gain-of-function and loss-of-function experiments show that these molecular gradients act instructively to determine synapse number. These patterns, in turn, fine-tune the perception of the stimulus and drive the behavioural response. Similar transcriptomic variation within neuronal types is observed in the vertebrate brain^7^ and may shape synapse number via gradients of cell recognition molecules acting through both genetically hard-wired programs and experience.

## Abstract (web summary)

Gradients of cell recognition molecules wire a sensorimotor interface in *Drosophila.*

## Main

Animals rely on visuomotor transformations to convert object locations in eye coordinates into directional movements^8^. The brain regions and neural circuits regulating this transformation in both vertebrates^9,10^ and invertebrates^11^ have been characterized. The precise neuronal connectivity underlying learned^12,13^ and innate visuomotor^14^ tasks is shaped by genetically hardwired mechanisms, experience or both.

**Fig. 1:**
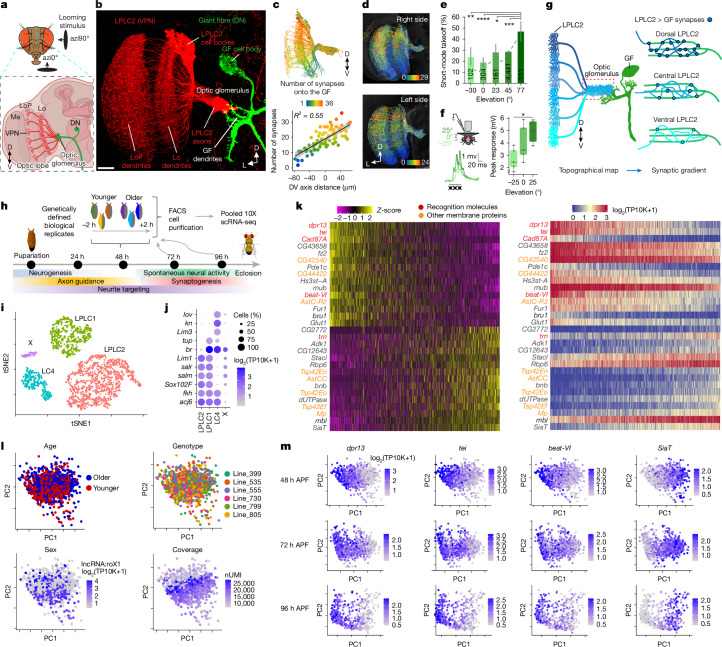
Molecular gradients correlate with synaptic gradients and behaviour. **a**, VPN dendrites cover the lobula (Lo) and LoP, and axons converge on optic glomeruli innervated by descending neurons (DNs). *n* = 12 brains. D, dorsal; V, ventral. The illustration of the brain was created using BioRender (https://biorender.com). **b**, Confocal projection of LPLC2 and the giant fibre (GF). *n*, individual brains. Scale bar, 20 μm. L, lateral. **c**, Connectomic reconstructions of LPLC2 neurons (‘hemibrain’^21^), coloured by LPLC2–GF synapse count (top), and linear regression of synapse number versus dorsoventral (DV) axis (bottom). The dots indicate individual neurons, and the error bands denote ±95% confidence intervals. The bottom panel was adapted from ref. ^5^, Springer Nature. **d**, Same as panel **c** (top), using FlyWire^22,23^. **e**, GF-mediated short-mode takeoffs in response to lateral (90°) looms at various elevations. The error bars denote ±95% confidence intervals. Numbers indicate total takeoffs (one per animal). Chi-squared test (*P* = 8.351 × 10^−7^) with post-hoc Bonferroni correction for multiple comparisons, **P = 0.0066 (−30° versus 77°), ****P < 0.0001 (0° versus 77°), *P = 0.0215 (23° versus 77°) and ***P = 0.000399 (45° versus 77°). **f**, GF response to looming at different elevations (left), and pooled peak GF responses across five trials (right). Dots denote individual flies (*n* = 5 biologically independent animals). The boxes denote quartiles, and the whiskers indicate 1.5× interquartile range. Repeated measures analysis of variance (rANOVA; *P* = 0.0048) with Sidak-adjusted post-hoc test, *P = 0.0385 (−25° versus 25°). **g**, Retinotopic maps transform into synaptic gradients between LPLC2 and GF without axonal retinotopy. **h**, Single-cell RNA sequencing (scRNA-seq) experimental design. The illustrations of the fly and pupa were adapted from ref. ^12^, Wiley Periodicals. **i**,**j**, tSNE plots of the 48 h APF dataset (**i**) with LPLC2, LPLC1 and LC4 annotated by marker gene expression (**j**). TP10K, transcripts per 10,000; X, unknown cell type. **k**, Heatmaps of the top 30 PC1 genes (15% variance explained) in LPLC2 at 48 h APF. Scaled expression levels (left), and log-normalized expression (right) are shown. **l**, LPLC2 neuron distributions along PC1–PC2 at 48 h APF, coloured by age, genotype, sex and coverage. lncRNA, long non-coding RNA; nUMI, number of unique molecular identifiers. **m**, PCA plots of LPLC2 neurons at 48 h APF, 72 h APF and 96 h APF, showing temporal expression changes in select genes from panel **k**. Source data

In flies, visuomotor transformation occurs between VPNs and descending neurons^15,16^ (Fig. 1a). VPNs include lobula columnar (LC) and lobula plate/lobula columnar (LPLC) neurons^4^ comprising approximately 30 cell types with 20–200 cells of each type per hemibrain^3,4^. For simplicity, we refer to these collectively as VPNs. The dendrites of each VPN type span 20–40° of visual space, forming a retinotopic feature-detecting map^17,18^ in the optic lobe. Their axons converge and terminate within VPN-type-specific optic glomeruli in the central brain, where they synapse onto the dendrites of descending neurons and other neurons (Fig. 1a). Most VPN types lose axonal retinotopy^4,5^, meaning spatially organized visual inputs onto VPN dendrites do not translate to ordered axonal projections.

We recently demonstrated that the transformation from visual input to motor output in VPN–descending neuron circuits relies on synaptic gradients, which form in most cases independent of axonal retinotopy^5^. We define synaptic gradients as connectivity patterns where the number of synapses from a presynaptic population (for example, a VPN type) to postsynaptic targets (for example, descending neurons) varies topographically along the anteroposterior or dorsoventral axes of visual space. This synaptic organization underlies directional behavioural responses^5^. Although these gradients originate from retinotopically guided dendritic inputs, the resulting axonal synaptic connections often encode visual space in a non-spatial, abstract manner. The molecular mechanisms underlying this transformation remain unclear.

To uncover the molecular basis of synaptic gradients, we examined a VPN type called LPLC2 neurons, a population of approximately 100 cells per hemibrain. Each LPLC2 neuron acts as a local looming detector, responding to dark, radially expanding motion centred on its receptive field^6,17^. The axons of LPLC2 neurons transmit visual information to the giant fibre (GF), a descending neuron that triggers a rapid looming-evoked escape takeoff^19,20^ (Fig. 1b). Previously, our analysis of two electron microscopy-based connectomic reconstructions^21–23^ revealed that LPLC2 neurons form a dorsoventral synaptic gradient, with dorsal LPLC2 neurons making more synapses with the GF than ventral neurons (Fig. 1c,d). Here we show that flies respond more strongly to dorsal than ventral looming stimuli correlating with the synaptic gradient.

To investigate the molecular basis of this gradient, we combined single-cell RNA sequencing (scRNA-seq), spatial transcriptomics and genetics with morphological, behavioural and physiological studies. We identified two cell recognition molecules expressed in a gradient across the LPLC2 population. Dpr13 regulates a gradient of synaptic inputs to the GF, and Beat-VI controls a synaptic gradient onto the dendrites of LPLC2. We demonstrate that varying levels of these proteins in LPLC2 specify different numbers of synaptic inputs and outputs. This represents a new mechanism regulating synaptic specificity.

## LPLC2–GF synaptic gradient guides escape

**Extended Data Fig. 1:**
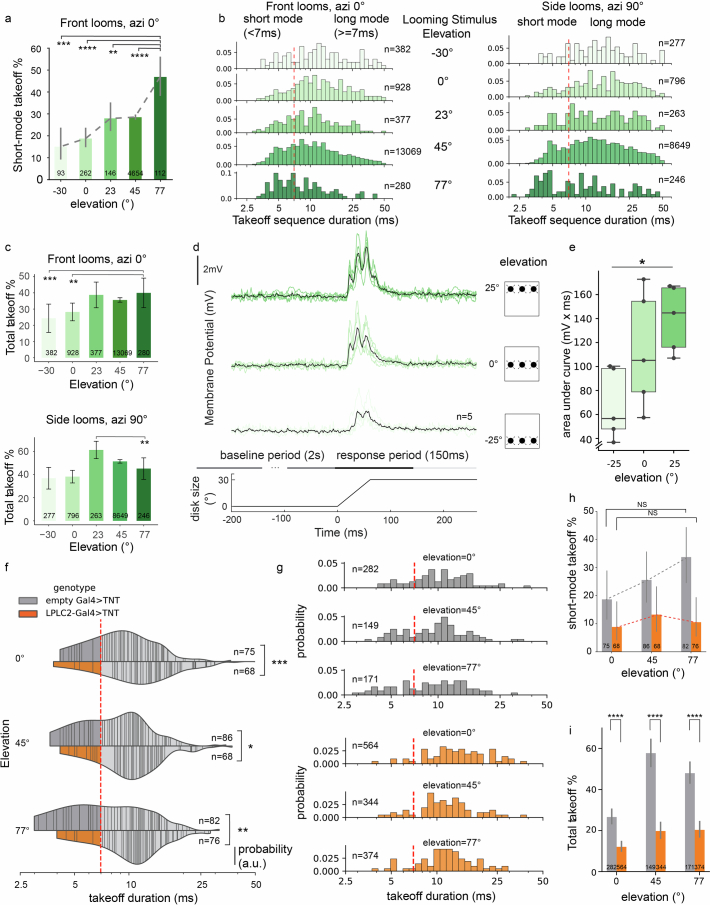
(related to Fig. 1). LPLC2-GF dorsoventral synaptic gradient biases the looming escape circuit towards short-mode takeoffs at higher stimulus elevations. **a**, GF-mediated short-mode (<7 ms sequence duration) takeoffs in response to frontal (0°) looms at various elevations. Error bars, ± 95% confidence intervals. Numbers, total takeoffs (one takeoff per animal). Chi-squared test (p < 0.0001) with post-hoc Bonferroni correction for multiple comparisons, ***P = 8.79 × 10^−4^ (−30° vs 77°), ****P = 0.03 × 10^−4^ (0° vs 77°), **P = 5.68 × 10^−3^ (23° vs 77°), ****P = 0.37 × 10^−4^ (45° vs 77°). **b**, Histograms showing the distribution of takeoff sequence durations in response to frontal (left) and lateral (right) looming stimuli at different elevations (detailed breakdown of data summarized in Fig. 1e). Short-mode and long-mode takeoffs are distinguished by the red dashed lines. Numbers, total trials. **c**, Total takeoff percentage in response to frontal (0°) and lateral (90°) looms at various elevations. Error bars, ± 95% confidence intervals. Numbers, total trials. Chi-squared test (P < 0.0001 for both azi=0° and azi=90°) with post-hoc Bonferroni correction for multiple comparisons, ***P = 2.46 × 10^−4^ (−30° vs 77°, azi=0°), **P = 0.0025 (0° vs 77°, azi=0°), **P = 0.0039 (23° vs 77°, azi=90°). **d**, Top: whole-cell electrophysiological recordings of the GF in response to looming stimuli at different elevations in control animals. Black traces represent averaged responses of five animals (corresponding to Fig. 1f), green traces represent responses of individual animals. Middle: baseline region (2 s before the onset of stimulus) and response region (150 ms after the onset of stimulus) defined in the traces for analysis of the GF responses. Bottom: change of disk size over time. **e**, Pooled mean of integrated potentials for the GF in response to looming stimuli at different elevations across five repeated trials. Each dot represents a single fly (n = 5 biologically independent animals). Boxes: quartiles; whiskers: 1.5× interquartile range. Repeated-measures one-way ANOVA (P = 0.01) with post-hoc Sidak correction for multiple comparisons, *P = 0.0496 (−25° vs 25°). **f**, Violin plots of takeoff sequence durations for lateral stimuli (90°) at different elevations (0°, 45°, 77°) in controls and LPLC2-silenced animals. Lines, single takeoffs. Short-mode and long-mode durations are separated by a red dashed line. n, total takeoffs. Mann-Whitney U test, ***P = 2.72 × 10^−4^ (0° elevation)) *P = 0.0118 (45° elevation), **P = 0.0040 (77° elevation). **g**, Histograms showing the distribution of takeoff sequence durations in response to lateral looming stimuli at different elevations in controls and LPLC2-silenced animals (detailed breakdown of **f**). Short-mode and long-mode takeoffs are distinguished by the red dashed lines. Numbers, total trials. **h**, Short-mode takeoff percentages at different elevations for controls and LPLC2-silenced animals. Error bars, ± 95% confidence intervals. Dashed lines indicated gradient trends. n, total number of takeoffs. Chi-squared test (^NS^P = 0.0977 for control; ^NS^P = 0.7058 for LPLC2-silenced). NS, not significant. **i**, Total takeoff percentages at different elevations for controls and LPLC2-silenced animals. Numbers, total trials. Error bars, ± 95% confidence intervals. Chi-squared test, ****P = 1.772 × 10^−7^ (0° elevation), ****P = 1.671 × 10^−16^ (45° elevation), ****P = 8.272 × 10^−11^ (77° elevation). Source data

To assess the function of the dorsoventral synaptic gradient between LPLC2 and the GF, we quantified escape responses to looming stimuli at different elevations. Flies take off in two modes: a faster short mode (featuring leg extension only, and taking the fly less than 7 ms to jump^24^) and a slower long mode (coordinated wing depression and leg extension^24^). Short-mode takeoffs are driven solely by the GF activation^20^. If LPLC2 neurons with more dorsal receptive fields form more synapses with the GF than ventral LPLC2 neurons, we predicted more short-mode takeoffs in response to higher-elevation stimuli. To test this hypothesis, we analysed thousands of previously collected^25^ high-speed videos of takeoffs elicited by looming stimuli at different elevations, and classified takeoffs by mode. The short-mode takeoff percentage increased as stimulus elevation changed from −30° to 77° (Fig. 1e and Extended Data Fig. 1a–c), as would be expected from higher engagement of the GF in response to more dorsal looming.

The GF receives direct visual input from only one other VPN type (LC4)^26^ in addition to LPLC2. LC4 neurons do not form a dorsoventral synaptic gradient with the GF^5^, suggesting the increase in GF-driven short-mode takeoffs at high elevations results directly from the LPLC2–GF synaptic gradient. We tested this by blocking synaptic transmission in LPLC2 and quantifying the looming responses in these flies (Extended Data Fig. 1f–h). Without LPLC2 input, the percentage of short-mode takeoffs did not change across elevations (Extended Data Fig. 1h), with the percentage of short-mode takeoffs greatly reduced at high (77°) elevations compared with control flies. To directly test whether LPLC2–GF connectivity biases GF activation, we performed in vivo whole-cell patch-clamp recordings in the GF during looming stimuli at three elevations. GF membrane depolarization was larger in response to dorsal than to ventral looming (Fig. 1f and Extended Data Fig. 1d,e). This provides direct evidence that the LPLC2–GF synaptic gradient generates a corresponding gradient of GF activation, ultimately shaping escape behaviour.

Electron microscopy reconstruction studies established that LPLC2 axons projections are not retinotopic (that is, intermingled) and yet form different numbers of synapses onto GF dendrites according to the location of the stimulus in the visual field to which they respond^4,5^ (Fig. 1g). We hypothesized that this pattern of synapses could be achieved through differential molecular recognition: individual LPLC2 neurons sampling different regions of visual space could express different levels of the same cell recognition molecule, which, in turn, would specify the number of synapses they form onto GF dendrites.

## Within-VPN-type molecular gradients

To investigate whether LPLC2 neurons exhibit molecular variation that correlates with the synaptic gradient, we examined the LPLC2 transcriptome at three developmental stages during and after synaptogenesis (48 h after puparium formation (APF), 72 h APF and 96 h APF; Fig. 1h). In addition to LPLC2, we profiled a related cell type, LPLC1, which forms synaptic gradients without axonal retinotopy with other descending neurons^5^, and LC4, a VPN type with axons arranged in a retinotopic manner^5^. As neuronal transcriptomes are highly dynamic during development and are affected by genetic background^27^, we introduced internal controls at each time point to account for transcriptional heterogeneity driven by these factors.

**Extended Data Fig. 2:**
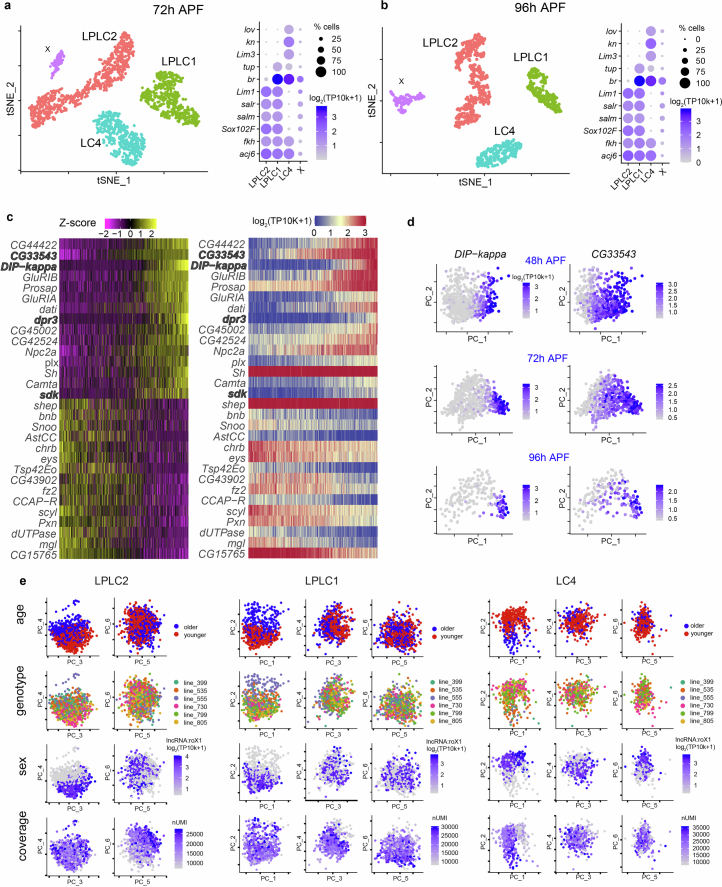
(related to Fig. 1). Synaptic gradients are associated with molecular heterogeneity across VPN cell types. **a-b**, t-SNE plots of 72 h APF (**a**) and 96 h APF (**b**) datasets. LPLC2, LPLC1, and LC4 neurons were annotated based on the expression of known transcription factors (right panels). X indicates unknown ectopic cell types. **c**, Heatmaps of the expression patterns of the top 30 genes with the highest contribution (loading) to differentially expressed genes along Principal Component 1 (PC1, 15.2% variance explained) across LPLC1 neurons at 48 h APF (see Fig. 1 legend for details). Genes encoding cell recognition molecules (IgSF superfamily) are highlighted in bold. **d**, PCA plots of LPLC1 neurons at 48, 72, and 96 h APF, colored by the expression levels of two cell recognition molecules from **c**. **e**, PCA plots of LPLC2, LPLC1, and LC4 neurons at 48 h APF colored by genotype, age (early vs late collection), genotype (DGRP line), sex (male-specific transcript), and coverage. PC1-6 for LPLC1 and LC4, and PC3-6 for LPLC2 are shown. Source data

We used genetic multiplexing to perform pooled single-cell profiling across biological replicates^27–29^ (including different genetic backgrounds and developmental stages; see Methods). Our dataset included approximately 600 high-quality single-cell transcriptomes per cell type and time point (Fig. 1i and Extended Data Fig. 2a,b). We validated the identity of each VPN cell type (LPLC2, LPLC1 and LC4) using known marker genes^27^ (Fig. 1j and Extended Data Fig. 2a,b). This provided approximately 30-times higher per-cell-type coverage than existing single-cell atlases of the *Drosophila* optic lobes^27,30^.

**Extended Data Fig. 3:**
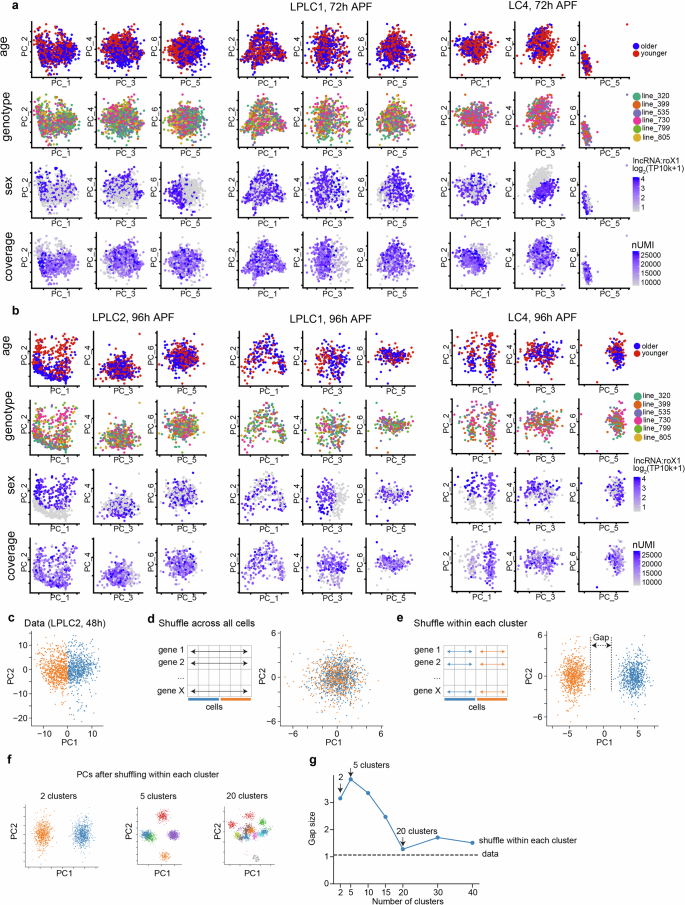
(related to Fig. 1). Further analysis of within-cell-type heterogeneity in gene expression across LPLC2, LPLC1 and LC4. **a-b**, PCA plots of LPLC2, LPLC1 and LC4 neurons at 72 h APF (**a**) and 96 h APF (**b**) colored by genotype, age (early vs late collection), genotype (DGRP line), sex (male-specific transcript), and coverage. Shown are PC1-6. Variance explained by PC1 at 72 h: 32% for LPLC2, 17% for LPLC1, 11% for LC4. Variance explained by PC1 at 96 h: 28% for LPLC2, 15% for LPLC1, 21% for LC4. **c-e**, Scatter plots of LPLC2 cells (48 h APF) embedded in the first two principal components (PC1 and PC2) colored by their cluster labels (n = 2, inferred from K-means clustering). PCs are calculated using the top 1000 highly variable genes based on the actual data (**c**), after shuffling gene expression levels for each gene independently across all cells (**d**), and after shuffling each gene only within each cluster (**e**), respectively. Shuffling gene expression across all cells (**d**) disrupts this gradient, indicating that the observed differences reflect coordinated gene expression rather than uncorrelated variation. Shuffling within each cluster (**e**) disrupts the internal gradient of each cluster, creating artificial gaps not present in the original data (**c**), which suggests that the continuous gradient observed in the original data cannot be represented as a set of discrete clusters. **f**, Scatter plots of cells in PCA embeddings after shuffling genes within each cluster for an increasing number of fine-grained clusters (n = 2,5,30). **g**, Gap size as a function of the number of clusters. Gap size is defined as the minimum distance in PC1 and PC2 space needed to connect 90% of cells into a single graph component. The gap size decreases and plateaus as the number of clusters increases. This means that a continuum can be approximated by a large number of discrete clusters. However, a small number of clusters (like 2) is insufficient to capture the continuous nature of the data. Source data

To explore heterogeneity in gene expression across each VPN cell type, we performed principal component analysis (PCA) separately for each cell type and time point (Fig. 1k–m and Extended Data Figs. 2c–e and 3a,b). At 48 h APF, PC1 captured genes expressed in a graded manner across difference LPLC2 neurons (Fig. 1k). For example, the most differentially expressed gene was *dpr13*, encoding a cell recognition protein of the immunoglobulin superfamily (IgSF)^31^. There was, however, no clear boundary separating neurons with high versus low *dpr13* expression levels. Many of the most differentially expressed genes also encode cell recognition molecules: IgSF (for example, *tei*, *beat-VI* and *dpr17*), leucine-rich-repeat (for example, *trn*) and cadherin (for example, *Cad87A*) families^31,32^ (Fig. 1k).

To test whether the transcriptional heterogeneity within LPLC2 neurons reflects discrete subtypes or a continuous gradient, we artificially clustered neurons and shuffled gene expression in two ways: across all cells or only within artificial clusters (Extended Data Fig. 3c–g). Shuffling across all cells disrupted the gradient, indicating that the observed variation arises from coordinated gene expression. Shuffling gene expression only within arbitrarily separated clusters of neurons introduced artificial gaps, showing that the original data do not naturally separate into discrete subtypes. Thus, the transcriptomic heterogeneity across LPLC2 neurons forms a continuous gradient.

The distribution of neurons along PC1 also did not correlate with developmental age, sex, genetic background or mRNA coverage (Fig. 1l and Extended Data Figs. 2e and 3a,b), indicating that molecular heterogeneity had a different origin. The graded expression of the top differentially expressed genes associated with PC1 at 48 h APF (*dpr13*, *beat-VI* and *tei* (encoding IgSF molecules), as well as *SiaT* (encoding sialyltransferase)) persisted through development (Fig. 1m).

In LPLC1 neurons, PC1 also captured gradients of IgSF-encoding transcripts (for example, *DIP-κ*, *CG33543*, *dpr3* and *sdk*) that persisted through development (Extended Data Fig. 2c,d). Similar to LPLC2, molecular gradients in LPLC1 could not be explained by any of the confounding factors (Extended Data Figs. 2e and 3a,b). By contrast, PC1 in LC4 correlated with technical factors (for example, transcript count per neuron; Extended Data Figs. 2e and 3a,b), indicating that its molecular heterogeneity was not biologically relevant.

In summary, we identified stable gradients of transcripts encoding recognition molecules in LPLC1 and LPLC2, but not in LC4, suggesting that molecular heterogeneity is a feature of VPNs forming synaptic gradients independent of axonal retinotopy^5^.

## Molecular and synaptic gradients match

**Fig. 2:**
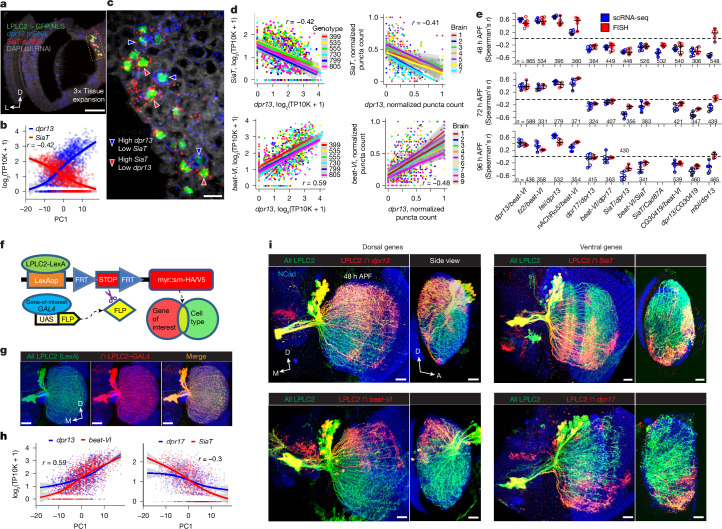
Gradients of recognition molecules align with synaptic gradients. **a**, Light-sheet projection of the *Drosophila* optic lobe showing LPLC2 nuclei and transcripts of *dpr13* and *SiaT*. *n* = 7 brains. Scale bar, 100 μm. **b**, Antiparallel expression of *dpr13* and *SiaT* across LPLC2 neurons (scRNA-seq; Fig. 1h–m). The smoothed lines indicate estimated mean expression trend. The error bands denote ±95% confidence intervals. *r*, Spearman’s rank correlation coefficient. **c**, Single 0.5-μm-thick slice from panel **a** (zoomed in region highlighted by the dashed rectangular box). The arrowheads denote individual LPLC2 neurons expressing markedly different levels of *dpr13* and *SiaT*. Scale bar, 10 μm. **d**, Comparison between scRNA-seq (left) and FISH (right) measuring correlation in expression for two pairs of graded genes: *dpr13–SiaT* (top) and *dpr13–beat-VI* (bottom) across LPLC2 neurons. The smoothed lines indicate linear regression fits, and the shaded bands denote ±95% confidence intervals. *r*, Spearman’s rank correlation coefficient (*r*_*s*_). **e**, Comparison of RNA-seq and HCR-FISH measuring correlation in expression for 12 pairs of genes at three developmental time points across LPLC2 neurons. Individual dots indicate *r*_*s*_ for each brain (FISH) and each genotype (scRNA-seq). The error bars denote means ± 95% confidence intervals. *n*, total neurons tested. **f**, Genetic approach to visualize a subset of neurons within a VPN cell type expressing a specific gene at a particular time point. **g**, Positive control for panel **f**. *n* = 5 brains (one side per animal tested). Scale bars, 20 μm. M, medial. **h**, Correlation between expression levels of *dpr13–beat-VI* and *dpr17–SiaT* (from scRNA-seq; Fig 1h–m), along PC1 across the LPLC2 population. **i**, Subsets of LPLC2 neurons expressing *dpr13*, *dpr17*, *beat-VI* and *SiaT*. *n*, number of brains (one side per animal tested); *n* = 11 for *dpr13*, *n* = 8 for *beat-VI*, *n* = 10 for *SiaT* and *n* = 7 for *dpr17*. Scale bars, 10 μm. A, anterior. For panels **a**–**d**,**h**–**I**, 48 h APF developmental time point. Data are from single experiments. Source data

**Extended Data Fig. 4:**
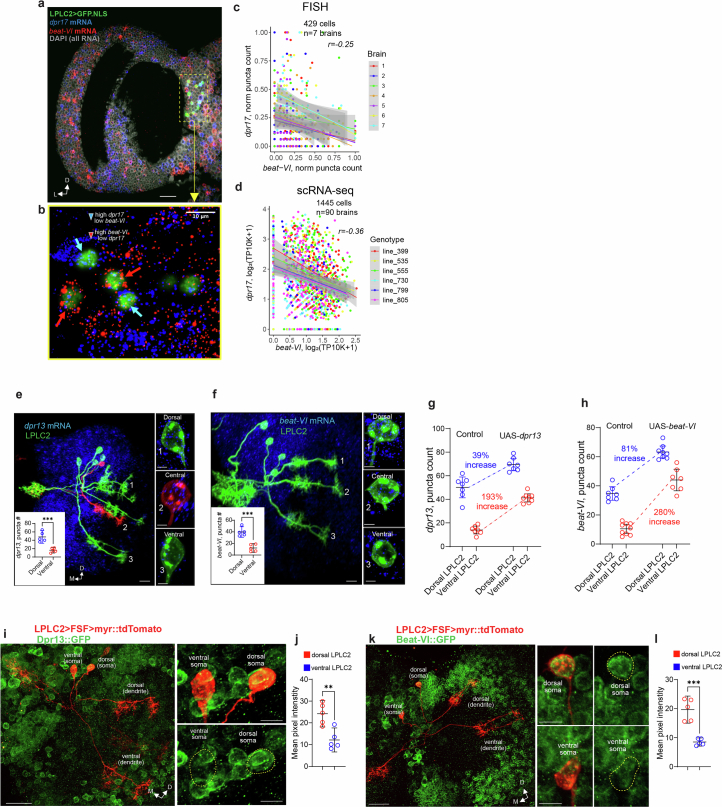
(related to Fig. 2). Molecular validation of graded gene expression in LPLC2. **a-b**, Light sheet projection of the expanded *Drosophila* optic lobe (48 h APF) with labeled LPLC2 nuclei and transcripts of *dpr17* and *beat-VI* (**a**) and a single slice (0.5 μm) from the light sheet projection (**b**, zoomed into the yellow dashed rectangular region); arrows indicate LPLC2 somas expressing markedly different levels of *dpr17* and *beat-VI*. n = 7 brains (one side per animal). Scale bar, 50 μm. **c-d**, Comparison of scRNA-seq (**c**) and HCR-FISH (**d**) measuring the correlation in expression for *dpr17* and *beat-VI* across LPLC2 neurons at 48 h APF. Smoothed lines: linear regression fits; shaded bands: ± 95% confidence intervals. r, Spearman’s rank correlation coefficient. **e-f**, Assessment of *dpr13* (**e**) and beat-VI (**f**) expression levels in sparsely labeled LPLC2 neurons using HCR-FISH. Left: representative images (scale bar, 20 μm). Right, cell bodies of dorsal, ventral, and central LPLC2 neurons (scale bar, 5 μm). Insets: comparison of *dpr13* (**e**) and *beat-VI* (**f**) puncta count in dorsal and ventral LPLC2 neurons. Circles represent single neurons (one per brain); data from one experiment. Error bars: mean ± 95% confidence intervals. Unpaired t-test with Welch’s correction (two-sided). **P = 0.002 for *dpr13*; ***P = 0.0002 for *beat-VI*. n = 5 neurons per location. **g-h**, Comparison of *dpr13* and beat-VI expression levels in dorsal and ventral LPLC2 neurons during UAS-*dpr13* (**g**) and UAS-*beat-VI* (**h**) overexpression in LPLC2. Circles, individual neurons (1-3 neurons per brain). The mean values were calculated for the control and experimental groups, and the percentage increase was reported. No statistical test was performed. Error bars, mean ± 95% confidence intervals. **i-l**, Assessment of Beat-VI and Dpr13 gradients at the protein level. Individual sparsely labeled LPLC2 neurons with dendrites sampling either dorsal, or ventral regions of the visual space, are co-localized with Dpr13 (**i**) and Beat-VI (**k**) constitutive protein traps (GFP-fusion proteins). GFP levels in dorsal vs ventral cell bodies are measured via mean pixel intensity (**j, l**). Circles represent individual neurons (n = 5 for each protein and each location). Error bars: means ± 95% confidence intervals. Unpaired t-test with Welch’s correction. **P = 0.00351 for Dpr13; ***P = 0.000221 for Beat-VI. Data from a single experiment. Scale bars, 20 μm and 5 μm on whole-cell and somata images, respectively. D, dorsal; L, lateral; M, medial. Source data

To verify gene expression gradients in LPLC2 neurons, we used single-molecule hairpin chain reaction fluorescent in situ hybridization^33^ (HCR-FISH), an epansion-assisted light-sheet microscopy (ExLSM)^34^ to visualize and count transcripts within LPLC2 neurons (Fig. 2a). The cell bodies of adjacent LPLC2 neurons exhibited striking differences in transcript levels of *dpr13* and *SiaT*, the two most differentially expressed genes in the LPLC2 dataset (Fig. 2b,c). Similar patterns were observed for *beat-VI* and *dpr17* (Extended Data Fig. 4a–d).

We quantified differential gene expression inferred from HCR-FISH analysis using Flyseg^35^, an automated volumetric instance segmentation algorithm that we previously developed (see Methods). The results of scRNA-seq and HCR-FISH were similar for genes exhibiting antiparallel (for example, *dpr13* and *SiaT*; Fig. 2d, top) and parallel expression patterns (for example, *dpr13* and *beat-VI*; Fig. 2d, bottom). Most of these relationships remained consistent throughout development (Fig. 2e and Supplementary Table 2), supporting our findings from scRNA-seq data. One exception was *muscleblind*(*mbl*); although this gene exhibited graded expression in scRNA-seq (Fig. 1k), it showed low, uniform expression in HCR-FISH. The reason for this discrepancy is unclear. In summary, most gene expression gradients across LPLC2 neurons from scRNA-seq data were confirmed by HCR-FISH.

**Extended Data Fig. 5:**
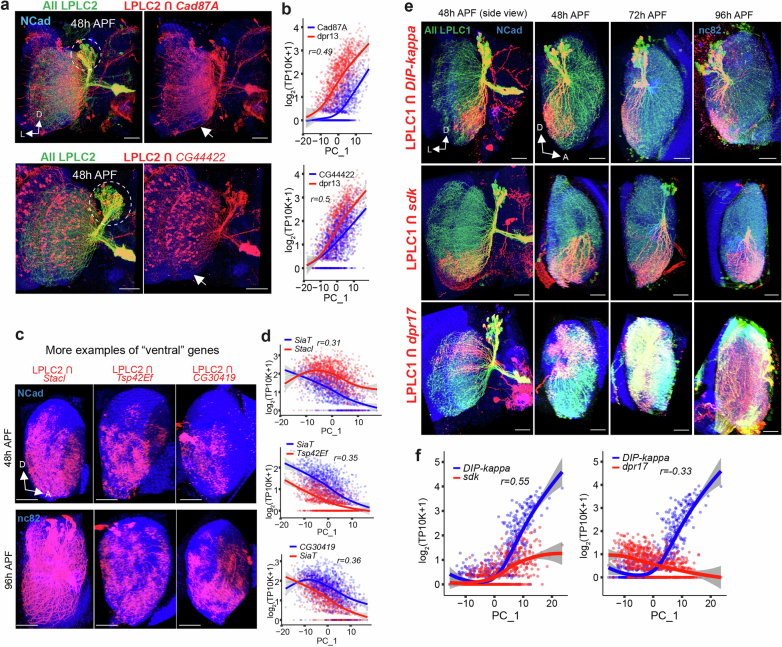
(related to Fig. 2). Retinotopic correlates of molecular gradients in LPLC2 and LPLC1. **a**, Subsets of LPLC2 neurons expressing *CG44422* and *Cad87A* at 48 h APF. White arrows indicate ventral regions of the lobula lacking expression of both *CG44422* and *Cad87A*. Dashed ovals, partial overlap of expression in somas, i.e., different LPLC2 neurons express different levels of *CG44422* and *Cad87A*. n = 4. **b**, Positive correlation between expression levels of *Cad87A*, *CG44422* and *dpr13* (inferred from scRNA-seq), along PC1 across the LPLC2 population at 48 h APF, indicating that both *CG44422* and *Cad87A* can be considered “dorsal” genes. **c**, Additional examples of genes expressed by ventral subpopulation of LPLC2 neurons. Red, dendrites of LPLC2 (lateral view of the lobula) that express stacl (n = 5 for 48 h and n = 3 for 96 h), *Tsp42Ef* (n = 4 for 48 h and n = 3 for 96 h) and *CG30419* (n = 6 for 48 h and n = 3 for 96 h). **d**, Positive correlation between expression levels of *stacl*, *Tsp42Ef* and *CG30419* (inferred from scRNA-seq), along PC1 across the *LPLC2* population at 48 h APF. **e**, Retinotopically biased gene expression across LPLC1 neurons. Red, subsets of LPLC1 neurons expressing *DIP*-*kappa*, *sdk* and *dpr17* across development (n = 9, 7, 6 for *DIP*-*kappa*; n = 8, 9, 5 for *sdk*; n = 4, 3, 3 for *dpr17*). **f**, Positive correlation between expression levels of *DIP*-*kappa* and *sdk* (top), and negative correlation between expression levels of *DIP*-*kappa* and *dpr17* (bottom), (from scRNA-seq, Fig. 1h–m), along PC1 across the LPLC1 population at 48 h APF, reflecting the retinotopically biased expression of these genes in **e**. n, brains (one side per animal). Scale bars, 20 μm. Panels **b, d, f**: Smoothed lines represent the estimated mean expression trend. Error bands: ± 95% confidence intervals. r, Spearman’s rank correlation coefficient. D, dorsal; L, lateral; A, anterior.

We next investigated whether there was a correlation between the retinotopic position of LPLC2 dendrites and gene expression levels. As the cell bodies of the LPLC2 neurons were arranged in a salt-and-pepper manner (Fig. 2c), we used a genetic intersection strategy to visualize the expression of transcriptional reporters for different genes in LPLC2 neurons in dendrites in different topographical locations in the visual field (Fig. 2f,g). Genes encoding recognition molecules *dpr13*, *beat-VI* and *Cad87A*, which showed correlated expression in scRNA-seq (Fig. 1k), were predominantly expressed by LPLC2 neurons with dendrites in the dorsal region of the lobula at 48 h APF (Fig. 2h,i and Extended Data Fig. 5a,b). Conversely, the expression of *SiaT*, *dpr17*, *CG03419*, *Tsp42Ef* and *stacl* was limited to the ventral region at the same developmental stage (Fig. 2h,i and Extended Data Fig. 5c,d). By combining reporter expression and HCR-FISH, we confirmed this heterogeneity, showing significantly higher *dpr13* and *beat-VI* transcript levels in dorsal LPLC2 neurons (Extended Data Fig. 4e,f). Heterogeneous expression of genes encoding recognition molecules in LPLC1 neurons had similar retinotopic correlates (Extended Data Fig. 5e,f), suggesting that such retinotopically biased expression gradients are a general feature of many VPN types.

To see whether these transcriptional gradients persist at the protein level, we used MIMIC-based protein traps^36^ generating GFP-tagged versions of two recognition proteins: Dpr13 and Beat-VI. This facilitated visualization of protein expression under their endogenous regulatory elements. Despite GFP accumulation in cell bodies (probably due to impaired trafficking), significant differences in GFP levels between dorsal and ventral LPLC2 neurons indicated that mRNA expression trends are maintained at the protein level (Extended Data Fig. 4i–l).

In summary, individual neurons of the same VPN type that sample different regions of visual space exhibit molecular heterogeneity. These neurons express gradients of recognition molecules that match the orientation of their synaptic gradients. Therefore, despite spatial intermingling, the axons retain distinct molecular identities. Next, we investigated the functional relevance of these molecular gradients.

## A Dpr13–DIP-ε gradient shapes escape

**Fig. 3:**
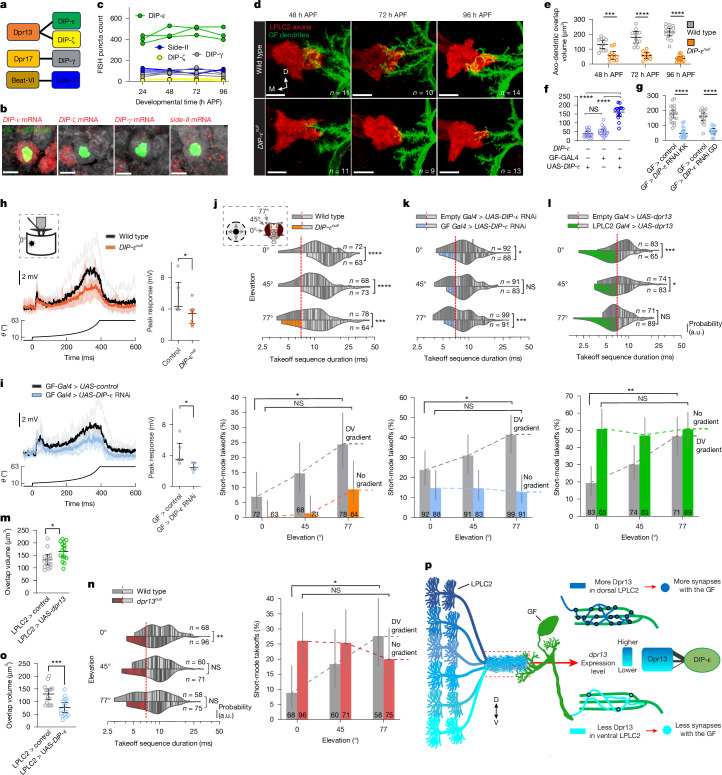
A gradient of DIP-ε–Dpr13 interactions controls a looming escape synaptic gradient. **a**, Molecular-binding partners of differentially expressed recognition molecules in LPLC2. **b**,**c**, Expression levels of candidate genes in the GF. Light-sheet projections of the GF nuclei (**b**), and quantification of their expression levels across development (**c**). The red puncta indicate candidate gene mRNA, and the circles denote individual GF neurons (one per animal; *n* = 3 neurons per gene). Scale bars, 10 μm. **d**, Confocal projections of colocalized LPLC2 axon terminals and the GF dendrites in wild-type and *DIP-ε*^*null*^ animals across development. *n*, brains (one side per animal); *n* = 11 and 11 for 48 h APF, *n* = 10 and 9 at 72 h APF, *n* = 14 and 13 at 96 h APF for wild type and *DIP-ε*^*null*^, respectively. Scale bars, 10 μm. **e**, LPLC2–GF axo-dendritic overlap in controls and *DIP-ε*^*null*^ across development. Unpaired *t*-test with Welch’s correction (two-sided). ***P = 0.000323 (48 h APF), ****P = 2.138 × 10^−5^ (72 h APF) and ****P = 3.344 × 10^−11^ (96 h APF). **f**, Same as panel **e** for DIP-ε rescue in the GF. One-way ANOVA (*F* = 63.753, *P* = 3.64 × 10^−13^) followed by Tukey’s (HSD) test for post-hoc pairwise comparisons. Not significant (NS) *P* = 0.588, ****P = 3.76 × 10^−12^ and ****P = 2.188 × 10^−11^. **g**, Same as panel **e** for controls and GF > *DIP-ε* RNAi animals. Unpaired *t*-test with Welch’s correction (two-sided). ****P = 1.347 × 10^−9^ and ****P = 7.587 × 10^−11^. **h**, Whole-cell patch-clamp recordings in the GF. GF responses to looming at *r*/*v* = 40 ms (left). Control (*n* = 5 flies) and *DIP-ε*^*null*^ (*n* = 7 flies) traces (individual and average) are overlayed. The looming stimulus profile over time is displayed below the GF responses. Quantification of expansion peak amplitudes from individual flies is also shown (right). *n*, biologically independent animals. The circles indicate mean values of two recordings per animal, and the error bars denote median and 25–75th percentiles. Mann–Whitney *U*-test *U* = 4, *P = 0.0303. **i**, Same as panel **h** for controls and GF > *DIP-ε* RNAi animals (*n* = 5 flies each). *U* = 2, *P = 0.03175. **j,** Violin plots of takeoff sequence durations for lateral stimuli at different elevations in wild-type and *DIP-ε*^*null*^ animals (top). The lines indicate single takeoffs. Short and long modes are separated by a red dashed line. *n*, total takeoffs. Mann–Whitney *U*-test, ****P = 2.763 × 10^−7^, ****P = 8.166 × 10^−9^ and ***P = 1.978 × 10^−4^. Short-mode takeoff percentages are also shown (bottom). The error bars denote mean ± 95% confidence intervals. The dashed lines indicate the wild-type DV gradient and its elimination *DIP-ε*^*null*^. Numbers refer to total takeoffs. Chi-squared test with post-hoc Bonferroni correction for multiple comparisons: *P = 0.0218 and NS *P* = 0.1149. **k**, Same as panel **j** for controls and GF > *DIP-ε* RNAi. *P = 0.031, NS *P* = 0.334 and ***P = 1.92 × 10^−4^ (top), and *P = 0.047 and NS *P* = 0.874 (bottom). **l**, Same as panel **j** for controls and LPLC2 > UAS-*dpr13*. ***P = 4.63 × 10^−4^, *P = 0.038 and NS *P* = 0.941 (top), and **P = 0.0018 and NS *P* = 0.8651 (bottom). **m**, Same as panel **e** for control animals, and LPLC2 > UAS-*dpr13* animals. Unpaired *t*-test with Welch’s correction (two-sided). *P = 0.022640. **n**, Same as panel **j** for wild type and *dpr13*^*null*^. **P = 0.0056, NS *P* = 0.4267 and NS *P* = 0.7714 (left), and *P = 0.0342 and NS *P* = 0.6221. a.u., arbitrary units. **o**, Same as panel **e** for control and LPLC2 > UAS-*DIP-ε* animals. Unpaired *t*-test with Welch’s correction (two-sided). ***P = 0.000503. **p**, The model shows that a DV gradient of Dpr13–DIP-ε interactions determines synapse number between individual LPLC2 neurons and the GF. Scale bars, 10 μm. In panels **e**–**g**,**m**–**o**, *n* values (images) and circles (plots) represent brains (one side per animal), and the error bars denote median ± 95% confidence intervals. Data are from single experiments. Source data

**Extended Data Fig. 6:**
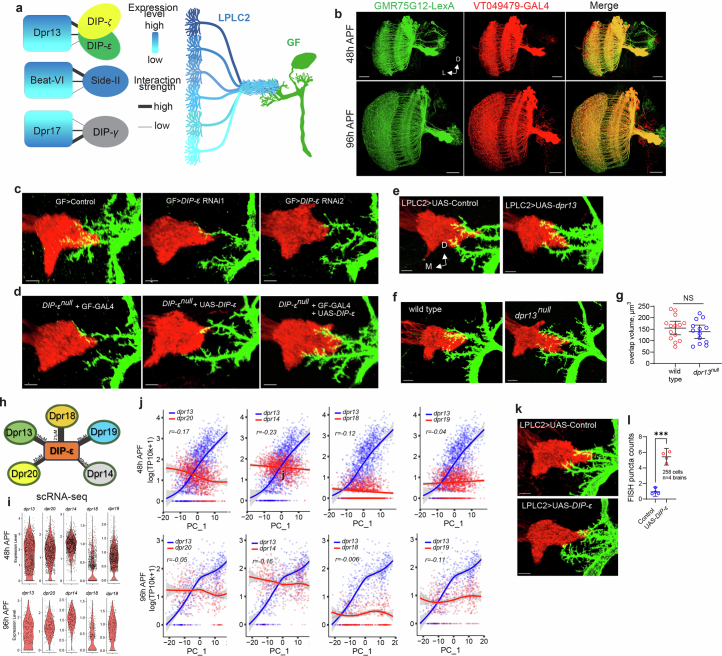
(related to Fig. 3). Synaptic gradient between LPLC2 and the GF is established through a gradient of DIP-ε::Dpr13 molecular interactions. **a**, Suggested model: to establish a synaptic gradient with LPLC2 based on dorsoventral expression gradient of any of the candidate molecules (Dpr13, Beat-VI, Dpr17), GF needs to express a molecular binding partner to recognize one or more of these molecules. **b**, Validation of VT049479-GAL4 expression in LPLC2 using GMR75G12-LexA as a reference. A complete overlap confirms that VT049479-GAL4 targets the entire LPLC2 population. n = 5 for 48 h APF, n = 4 for 96 h APF. Scale bars, 20 μm. **c**, Confocal projections of LPLC2 axon terminals and the GF dendrites in wild-type animals, as well as animals expressing control RNAi and two different *DIP-ε* RNAi in the GF (n = 19, 19, 11). **d**, Same as **c** for the DIP-*ε* rescue experiment (overexpression of DIP-ε cDNA in the GF in *DIP-*^*null*^ background). n = 14, 16, 13. **e**, Same as **c** for control animals and animals overexpressing *dpr13* in LPLC2. n = 14, 15. **f**, Same as **c** for control and *dpr13*^*null*^ animals. n = 14, 13. **g**, LPLC2-GF axo-dendritic overlap in control and *dpr13*^*null*^ animals. Circles, brains (one side per animal). Error bars: means ± 95% confidence intervals. Unpaired t-test with Welch’s correction (two-sided). ^NS^P = 0.405. NS, not significant. **h**, Protein interaction map showing binding strength (affinity values are inversely proportional to edge thickness) between DIP-ε and multiple Dpr paralogs expressed in LPLC2. **i**, Expression levels of genes encoding DIP-ε binding Dpr paralogs in LPLC2 at 48 h and 96 h APF (inferred from scRNA-seq data generated in this study). **j**, Correlation between expression levels of genes encoding DIP-ε binding Dprs in LPLC2 at 48 h and 96 h APF (inferred from scRNA-seq), along PC1 across the LPLC2 population at 48 h and 96 h APF. Smoothed lines represent the estimated mean expression trend. Error bands: ± 95% confidence intervals. r, Spearman’s rank correlation coefficient. **k**, Same as **c** for control animals and animals overexpressing *DIP-ε* in LPLC2. n = 15, 15. **l**, FISH puncta count across LPLC2 neurons in controls and animals overexpressing *DIP-ε* in LPLC2. Circles, averaged values across all LPLC2 neurons per hemibrain. Error bars: mean ± 95% confidence intervals. Unpaired t-test with Welch’s correction (two-sided). ***P = 0.0001. Panels **b**-**f**, **k**: n, brains (one side per animal tested); data represent single experiments. Panels **b**-**f**, **k**: scale bars, 5 μm. D, dorsal; L, lateral; M, medial. Source data

LPLC2 neurons express genes encoding IgSF recognition proteins in a dorsoventral gradient, with *dpr13* and *beat-VI* having higher expression levels in dorsal LPLC2 neurons and *dpr17* having higher expression levels in ventral neurons. We hypothesized that one or more of these molecular gradients could specify the gradient in synapse number between LPLC2 axons and GF dendrites (Extended Data Fig. 6a,b). If this were the case, the GF would need to express cell-surface proteins that bind to one or more of these three recognition molecules. This would allow the differential molecular expression in LPLC2 neurons to be converted into differential cell adhesion between individual LPLC2 neurons and the GF. Dpr proteins bind to DIP proteins, a related but different IgSF subfamily^31^. There are multiple paralogues of each, and interactions between them have been characterized^37^. Similarly, Beat proteins bind to Sides, which are also IgSF members^31,38^ (Fig. 3a). As a step towards testing our hypothesis, we assessed expression levels of genes encoding binding partners of Dpr13, Beat-VI and Dpr17 (DIP-ε, Side-II and DIP-γ, respectively) in the GF using HCR-FISH. *DIP-ε*, encoding a binding partner of Dpr13, was the only gene with strong expression in the GF during development (Fig. 3b,c).

To determine whether DIP-ε regulates synaptic connections between LPLC2 axons and GF dendrites, we explored the interaction between them in DIP-ε-deficient flies. Animals with no DIP-ε (*DIP-ε*^*null*^ allele) displayed an approximately tenfold reduction in axo-dendritic overlap (Fig. 3d,e). Knockdown of *DIP-ε* specifically in the GF using two different RNA interference (RNAi) lines also significantly reduced this overlap (Fig. 3f and Extended Data Fig. 6c). Wild-type levels of overlap were restored in *DIP-ε*^*null*^ flies through targeted expression of *DIP-ε* in the GF (Fig. 3f and Extended Data Fig. 6d). Thus, DIP-ε promotes interaction between LPLC2 axons and GF dendrites.

**Extended Data Fig. 7:**
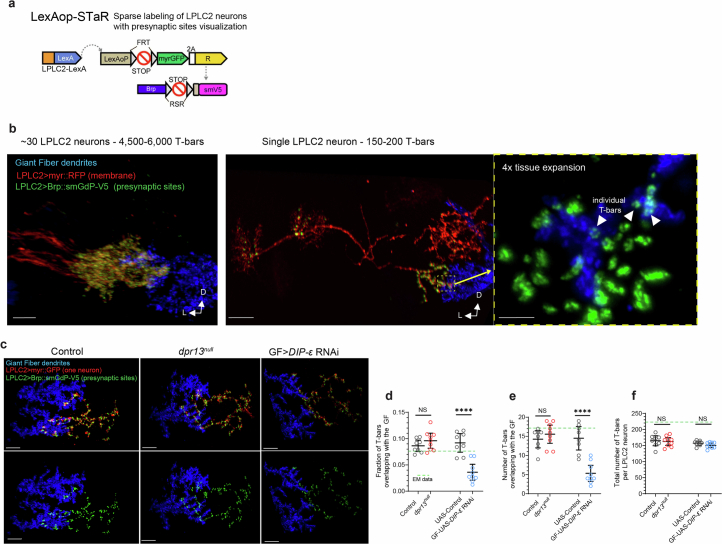
(related to Fig. 3). Analysis of LPLC2-GF connections using synaptic labeling. **a**, Genetic strategy (LexAop-STaR, **S**ynaptic **Ta**gging with **R**ecombination) for generating sparsely labeled clones of LPLC2 neurons (with a fluorescent membrane marker) and visualizing their presynaptic sites (T-bars) with Brp-smGdP-V5. Adapted from ref. ^5^, Springer Nature. **b**, LPLC2 neurons labeled using LexAop-STaR. Left: confocal projection of LPLC2 glomerulus (axon terminals of ~30 LPLC2 neurons, labeled using long heat shock) co-localized with the GF dendrites (n = 6; scale bar, 5 μm). Middle: light sheet projection of a single LPLC2 neuron, imaged with 4x tissue expansion (n = 4; scale bar, 20 μm). Right: high-resolution image of the magnified view of presynaptic sites (T-bars) of a single LPLC2 neuron, co-localized with the GF dendrites (n = 4; scale bar, 1 μm). White arrows indicate individual T-bars, characterized by their distinctive ring-like shape and a typical diameter of 200–250 nm. n, brains (one neuron/optic glomerulus per brain). **c**, Same as **b**. Left to right: control animals (n = 8), *dpr13*^*null*^ animals (n = 9), and animals expressing *DIP-ε* RNAi in the GF (n = 9). n, brains (one neuron per brain). Scale bars, 5 μm. **d**, Fraction of T-bars per single LPLC2 neuron overlapping with the GF dendrites. Left: control vs. *dpr13*^*null*^; right: control vs. GF > UAS-*DIP-ε* RNAi. Dots represent single neurons. Error bars: mean ± 95% confidence intervals. Unpaired t-test with Welch’s correction (two-sided). ^NS^P = 0.239; ****P = 0.000047. NS, not significant. **e**, Same as in **d**, measuring the number of T-bars overlapping with the GF dendrites. ^NS^P = 0.372; ****P = 0.00003. **f**, Same as in **d**, measuring the total number of T-bars per single LPLC2 neuron. ^NS^P = 0.785; ^NS^P = 0.125. In **d–f**, green lines indicate corresponding values inferred from the hemibrain connectome reconstruction. In **d**, the discrepancy between connectome-based and anatomy-based values likely reflects additional T-bars in the lobula/lobula plate not included in this analysis. Panels **d-f**: data from a single experiment. D, dorsal; L, lateral. Source data

To assess whether this effect was associated with a decrease in synapse number, we visualized presynaptic sites (marked with T-bar-associated endogenously tagged Bruchpilot (Brp) protein) in sparsely labelled LPLC2 neurons using a modification of the synaptic tagging with recombination (STaR)^39^ technique (Extended Data Fig. 7a,b). RNAi knockdown of *DIP-ε* in the GF led to a significant reduction in the number of LPLC2 T-bars contacting the GF dendrites (Extended Data Fig. 7c–f). Thus, DIP-ε is required in the GF to establish synaptic connectivity with LPLC2 axons.

**Extended Data Fig. 8:**
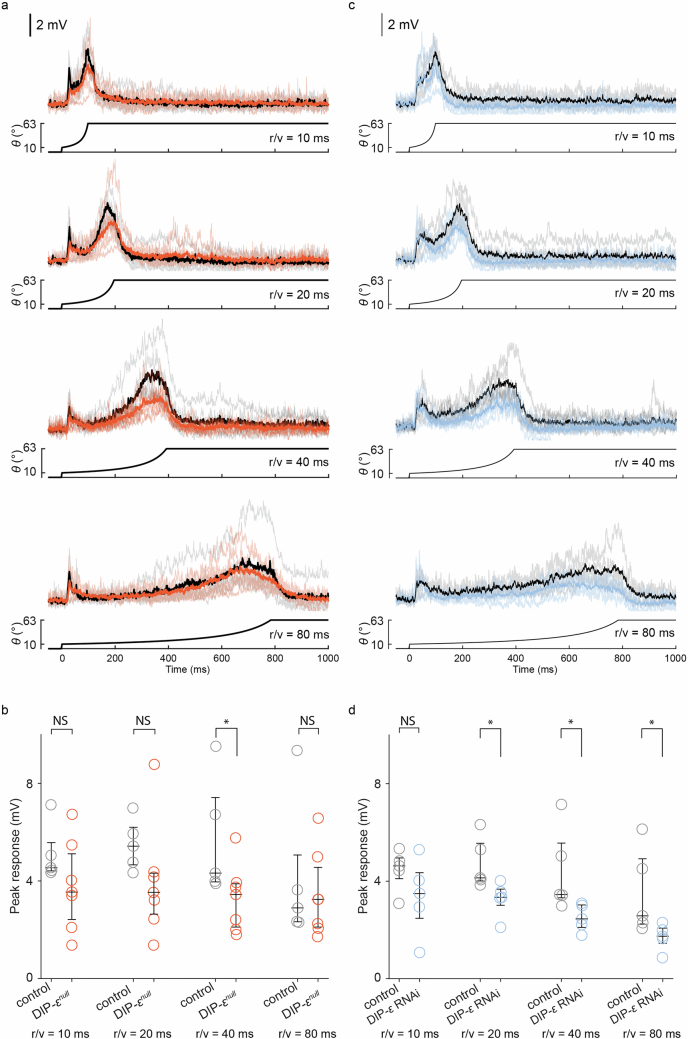
(related to Fig. 3). Electrophysiology of the GF. **a**, GF responses to looming stimuli, in r/v = 10, 20, 40 and 80 ms. Control (average: black, individual fly: grey) and *DIP-ε*^*null*^ (average: orange, individual fly: light orange) traces are overlayed. Looming stimulus profile over time is displayed below the GF responses. n = 5 for controls; n = 7 for *DIP-ε*^*null*^ flies. **b**, Quantification of expansion peak amplitudes in **a** from individual flies. n, biologically independent animals; circles, mean values of two recordings per animal. Error bars: median and 25^th^/75^th^ percentiles. Mann-Whitney U test. r/v = 10 ms: U = 8, ^NS^P = 0.149. r/v = 20 ms: U = 6, ^NS^P = 0.07323. r/v = 40 ms: U = 4, *P = 0.0303. r/v = 80 ms: U = 14, ^NS^P = 0.6389. **c**, Same as **a** for controls (grey) and animals overexpressing *DIP-ε* RNAi in the GF (light blue). n = 5 for controls and *DIP-ε* RNAi. **d**, Quantification of expansion peak amplitudes in **c** from individual flies. n, biologically independent animals (two trials per animal); circles, mean values of two recordings per animal. Error bars: median and 25^th^/75^th^ percentiles. Mann-Whitney U test. r/v = 10 ms: U = 6, ^NS^P = 0.222. r/v = 20 ms: U = 1, *P = 0.01587. r/v = 40 ms: U = 2, *P = 0.03175. r/v = 80 ms: U = 2, *P = 0.03175. Source data

**Extended Data Fig. 9:**
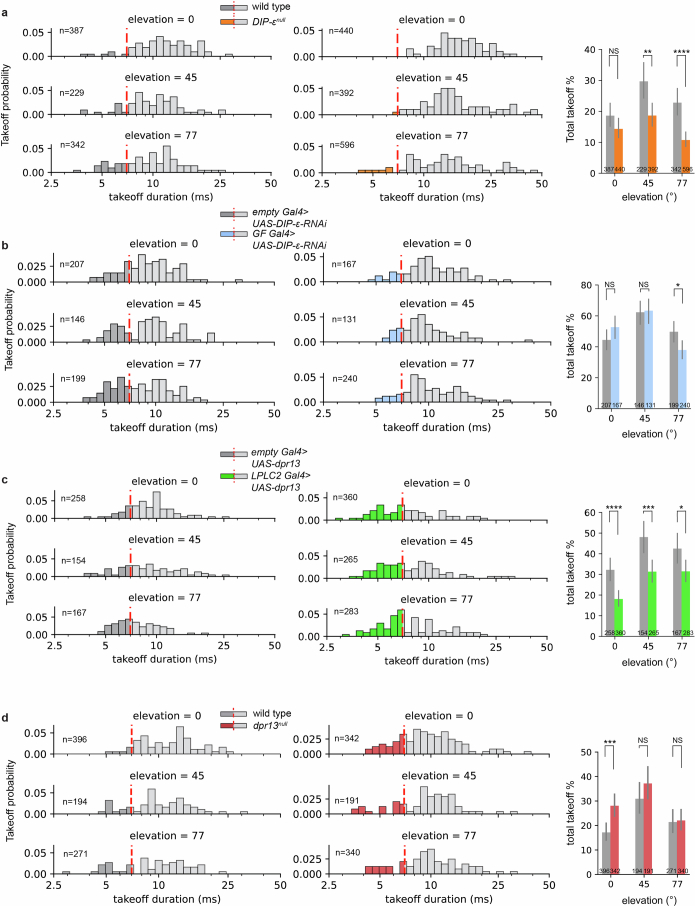
(related to Fig. 3). Effects of Dpr13 and DIP-*ε* on GF-mediated takeoff behavior. **a**, Left, Histograms showing the distribution of takeoff sequence durations at different stimulus elevations (0°, 45°, and 77°) in wild-type and *DIP-ε*^*null*^ flies. Short-mode and long-mode takeoffs are distinguished by red dashed lines. n, number of trials. Right, total takeoff percentages at different elevations for wild-type and *DIP-ε*^*null*^ flies. Error bars, ± 95% confidence intervals. Numbers, total number of trials. Chi-squared test. ^NS^P = 0.1164 (0° elevation), **P = 0.0021 (45° elevation), ****P = 1.122 × 10^−6^. (77° elevation) NS, not significant. **b**, Same as **a** for controls and flies expressing *DIP-ε* RNAi in the GF. Error bars, ± 95% confidence intervals. Numbers, total number of trials. ^NS^P = 0.1380 (0° elevation), ^NS^P = 0.9581 (45° elevation), *P = 0.0167 (77° elevation). **c**, Same as **a** for controls and flies overexpressing *dpr13* in LPLC2 neurons. Error bars, ± 95% confidence intervals. Numbers, total number of trials. ****P = 7.523 × 10^−5^ (0° elevation), ***P = 9.44 × 10^−4^ (45° elevation), *P = 0.0234 (77° elevation). **d**, Same as **a** for controls and *dpr13*^*nul*l^ flies. Error bars, ± 95% confidence intervals. Numbers, total number of trials. ***P = 5.35 × 10^−4^, (0° elevation) ^NS^P = 0.2358 (45° elevation), ^NS^P = 0.9229 (77° elevation). Source data

If DIP-ε is required to form functional LPLC2–GF synapses, then we would expect loss of DIP-ε to reduce the GF responses and to disrupt short-mode takeoffs. We also anticipated that, if too few LPLC2–GF synapses formed to make a dorsoventral gradient, then the number of short-mode takeoffs would no longer increase with increasing elevation of the looming stimulus. To test these assumptions, we first performed whole-cell patch-clamp recordings in the GF while presenting looming stimuli to both *DIP-ε*^*null*^ and control flies. As predicted, we observed a reduction in the peak magnitude of the GF response in *DIP-ε*^*null*^ animals and in those expressing *DIP-ε*RNAi in the GF compared with controls (Fig. 3h,i and Extended Data Fig. 8). *DIP-ε*^*null*^ and DIP-ε-RNAi-expressing animals also had dramatically reduced short-mode takeoffs across all stimulus elevations and a smaller difference between 0° and 77° than controls (Fig. 3j,k and Extended Data Fig. 9a,b). These results support a role for DIP-ε in the GF in establishing graded synaptic connectivity with LPLC2 neurons.

We next tested the causal role of the Dpr13–DIP-ε gradient on takeoff behaviour. We did this in two ways. First, we sought to retain strong LPLC2–GF connectivity while altering the connectivity gradient. To do this, we increased the level of *dpr13* uniformly across the LPLC2 population (heretofore referred to as ‘overexpression’, that is, superimposed on the endogenous *dpr13* gradient). This disproportionately increased *dpr13* mRNA expression in ventral LPLC2 neurons (193% in ventral versus 39% in dorsal; Extended Data Fig. 4g). Compared with controls, animals overexpressing *dpr13* in LPLC2 exhibited a higher percentage of short-mode takeoffs (Fig. 3l and Extended Data Fig. 9c). This gain of function was most pronounced at lower (0°), decreased at medium (45°) and was absent at higher (77°) stimulus elevations, resulting in no significant change in short-mode escape frequency across elevations (that is, animals responded uniformly to dorsal and ventral looming stimuli). Thus, when the *dpr13* gradient was flattened, the dorsoventral gradient of short-mode takeoffs was eliminated (Fig. 3l). This manipulation also led to a modest increase in LPLC2–GF axo-dendritic overlap (Fig. 3m and Extended Data Fig. 6e). Our data support a causal relationship between the level of *dpr13* and the number and graded distribution of LPLC2 synapses onto the GF.

In a second series of experiments, we examined the consequences of removing Dpr13. *dpr13*^*null*^ flies showed no reduction in the overlap between LPLC2 axons and GF dendrites or the number of LPLC2 T-bars contacting the GF dendrites (Fig. 3m and Extended Data Figs. 6f,g and 7c–f). This may be due to redundancy, as LPLC2 neurons also express four other Dprs that bind to DIP-ε (*dpr14*, *dpr18*, *dpr19* and *dpr20*; Extended Data Fig. 6h,i). To test this, we ectopically expressed *DIP-ε* in LPLC2, promoting *cis*-interactions that block *trans*-interactions with the GF (an effect observed for other DIP–Dpr pairs^40,41^). This manipulation reduced LPLC2–GF overlap (Fig. 3o and Extended Data Fig. 6k,l), mimicking the phenotype of DIP-ε removal from the GF.

Of the Dpr paralogues that bind to DIP-ε, only Dpr13 is expressed in a graded manner (Extended Data Fig. 6j). Although *dpr13*^*null*^ flies displayed no changes in the LPLC2–GF axo-dendritic overlap, they no longer maintained the dorsoventral gradient of short-mode takeoffs (Fig. 3n and Extended Data Fig. 9d). This mainly resulted from a significantly increased short-mode takeoff percentage at lower stimulus elevations. The reason for this is unknown. However, it raises the possibility that it is not the absolute level of Dpr13 expression that determines synapse number, but rather the relative differences in expression levels between different LPLC2 neurons.

In summary, DIP-ε and Dpr13 function as a ligand–receptor pair to regulate LPLC2–GF synaptic connectivity. Our findings support a model in which the gradient of Dpr13–DIP-ε interactions shapes the synaptic gradient (Fig. 3p).

## A Beat-VI–Side-II gradient shapes tuning

**Extended Data Fig. 10:**
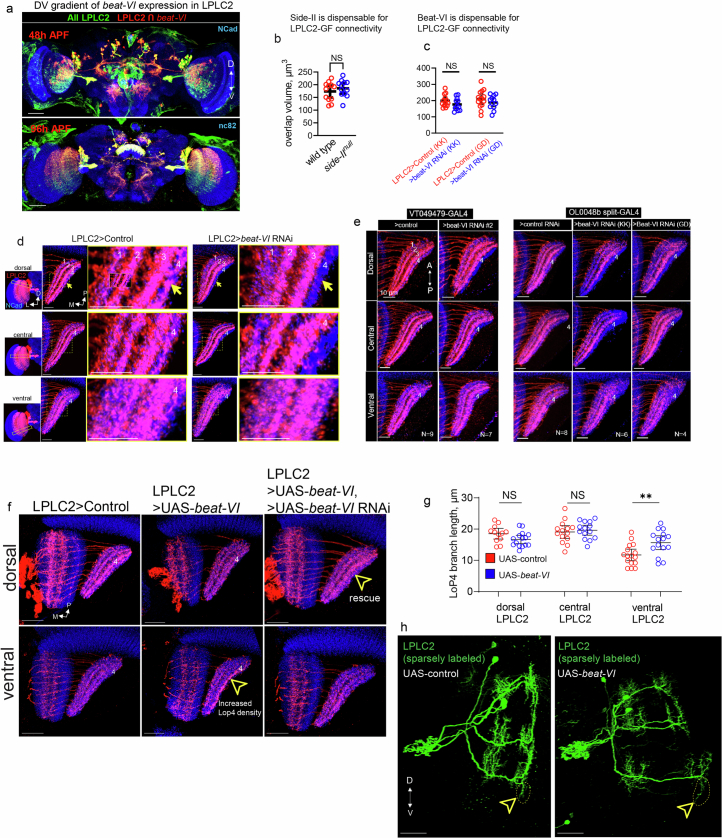
(related to Fig. 4). Graded expression of *beat-VI* across LPLC2 neurons differentially affects LoP4 dendritic wiring. **a**, Expression of *beat-VI* is biased towards the dorsal part of LPLC2 population. Red, subsets of LPLC2 neurons expressing *beat-VI* at 48 h APF (top) and 96 h APF (bottom). Green, all LPCL2 neurons. n = 8 brains at 48 h APF; n = 4 brains for 96 h APF. Scale bar, 50 μm. **b-c**, Comparison of axo-dendritic overlap between LPLC2 and the GF in controls (n = 12) vs *side-II*^*null*^ (n = 12) animals (**b**), and in animals expressing control RNAi (n = 13, 14) vs two *beat-VI* RNAi (n = 12, 12) in LPLC2 (**c**). Circles, brains (one side per animal). Error bars: mean ± 95% confidence intervals. Unpaired t-test with Welch’s correction (two-sided). ^NS^P = 0.3884 (control vs *side-II*^*null*^); ^NS^P = 0.149 (UAS-control vs UAS-*beat-VI* RNAi KK); ^NS^P = 0.2956 (UAS-control vs UAS-*beat-VI* RNAi GD). NS, not significant. **d**, Confocal projections of LPLC2 dendrites (entire LPLC2 population labeled) in the lobula plate in control animals (n = 16) and in animals with *beat-VI* RNAi expressed in LPLC2 (n = 18). Left panels: posterior views of the LPLC2 population with dashed rectangles indicating the location of cross-sections for dorsal, central, and ventral subsets of LPLC2. Numbers, LoP layers. Yellow arrows, LoP4 layer. Scale bars, 10 μm. **e**, Same as **d** for two different LPLC2 GAL4 driver lines and two different *beat-VI* RNAi lines. **f**, Confocal projections of LPLC2 dendrites (entire LPLC2 population labeled, dorsal and ventral cross-sections compared) in the lobula plate in control animals (left, n = 4), animals overexpressing *beat-VI* cDNA (middle, n = 4), and animals overexpressing *beat-VI* cDNA while also expressing *beat-VI* RNAi (right, n = 3). Yellow arrowheads indicate changes in LoP4 dendritic density. Scale bars, 20 μm. **g**, Length of LoP4 dendritic branches for dorsal, central and ventral LPLC2 neurons in control (n = 12, 14, 16 dorsal, central, and ventral) and LPLC2 > UAS-*beat-VI* (n = 15, 14, 12 dorsal, central, and ventral) flies. n, individual neurons (one cell per brain). Dots, individual neurons. Error bars: mean ± 95% confidence intervals. Unpaired t-test with Welch’s correction (two-sided). ^NS^P = 0.073; ^NS^P = 0.643; **P = 0.006. **h**, Confocal projections of sparsely labeled LPLC2 neurons. Representative images of control animals (left) and animals overexpressing *beat-VI* in LPLC2 (right). Differences in the length of LoP4 dendritic branches across ventral LPLC2 neurons are highlighted. Scale bars, 20 μm. Panels **d**–**f, h**: data represent single experiments. Panels **d**-**f**: n, brains (one side per animal). D, dorsal; V, ventral; P, posterior; M, medial; L, lateral. Source data

We next investigated the function of other genes displaying dorsoventral expression gradients in LPLC2 neurons (Fig. 1k). *beat-VI*, encoding another IgSF recognition molecule^38^, exhibited a dorsoventral gradient, with higher expression in dorsal and lower expression in ventral LPLC2 neurons (Fig. 2i and Extended Data Fig. 10a). Neither the loss of Beat-VI nor its binding partner Side-II affected LPLC2–GF axo-dendritic overlap (Extended Data Fig. 10b,c). Given that LPLC2 dendrites also receive graded synaptic inputs (see below), we tested whether *beat-VI* controls this gradient.

**Fig. 4:**
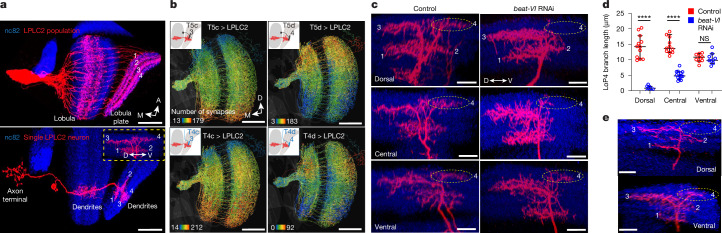
A gradient of Beat-VI–Side-II interactions controls a dendritic synaptic gradient between T4d/T5d and LPLC2 neurons. **a**, Confocal projections of the LPLC2 population (top; *n* = 8 brains) and an individual LPLC2 neuron (bottom; *n* = 11 neurons, one per brain), highlighting the dendritic branches in the LoP. Numbers refer to LoP layers. The inset shows the posterior view of a single LoP dendrite with branches extending into one of the four cardinal directions in each layer. **b**, In the connectomic reconstructions (FlyWire^22,23^) of LPLC2 neurons, the skeleton of each neuron is colour coded by the number of inputs each LPLC2 neuron receives from T4c or T4d and T5c or T5d neurons. The insets show schematics of single LPLC2 (red) and T4c or T4d or T5c or T5d neurons, overlaid on the lobula or LoP outlines. Scale bars, 50 μm (**a**,**b**). **c**, Confocal projections of LoP dendrites (posterior view) in individual dorsal, central and ventral LPLC2 in control and LPLC2 > *beat-VI* RNAi flies. The numbers denote LoP layers. The dashed ovals indicate LoP4 dendritic branches. *n*, neurons, one per brain (*n* = 12 and *n* = 9 for dorsal control versus *beat-VI* RNAi; *n* = 11 and *n* = 13 for central control versus RNAi; *n* = 12 and *n* = 9 for dorsal control versus RNAi; and *n* = 9 for ventral control versus RNAi). **d**, Length of LoP4 dendritic branches for dorsal, central and ventral LPLC2 neurons in control and LPLC2 > *beat-VI* RNAi flies. The circles denote neurons (one neuron per brain), and the error bars indicate median ± 95% confidence intervals. Unpaired *t*-test with Welch’s correction. ***P = 2.95 × 10^−8^ (dorsal LPLC2), ****P = 2.264 × 10^−8^ (central LPLC2) and NS *P* = 0.923 (ventral LPLC2). **e**, Confocal projections of individual LoP dendrites (posterior view) of dorsal and ventral LPLC2 neurons in T4/T5 > *side-II* RNAi flies. The dashed ovals denote LoP4 dendritic branches. *n* = 6 neurons for both positions (one neuron per brain). Scale bars, 10 μm (**c**,**e**). Data are from single experiments (**a**,**c**,**e**). Source data

Each LPLC2 neuron has four dendritic branches in the lobula plate (LoP), one in each layer, extending in four cardinal directions and corresponding to motion sensitivity in each layer^6^ (Fig. 4a, bottom). The response measured at the LPLC2 axon is a non-linear sum of the activity in each of these four branches. Individual LPLC2 neurons thus serve as local looming detectors. They respond most strongly to looming stimuli originating at their receptive field centre extending radially outwards and simultaneously generating motion in all four cardinal directions^6^. Using the FlyWire^22,23^ connectome, we found that two of the four classes of T4/T5 neurons, T4c/T5c and T4d/T5d, form antiparallel dorsoventral synaptic gradients onto LPLC2 dendrites in LoP3 and LoP4 layers, respectively (Fig. 4b).

We and others have shown that Beat-VI regulates synaptic specificity in the *Drosophila* motion detection system^42,43^. Beat-VI and its interacting partner Side-II^38^ are required for matching downwards motion-detecting T4d/T5d neurons with their postsynaptic partners (for example, LLPC3 and VS neurons) in LoP4 (refs. ^42,43^). Because LPLC2 is part of this circuit, we hypothesized that the Beat-VI gradient might selectively regulate the inputs of T4/T5d neurons to the dendrites of LPLC2 in LoP4.

We next sought to test whether the Beat-VI gradient regulates the gradient of synaptic inputs to LPLC2 in LoP4; that is, the dorsal and ventral LPLC2 neurons may receive different amounts of upwards and downwards motion information, potentially linked to the Beat-VI levels. Supporting this, RNAi knockdown of *beat-VI* altered LPLC2 dendritic morphology in a location-dependent manner. LoP4 branches were missing in dorsal LPLC2, moderately affected in central LPLC2 and unaffected in ventral LPLC2 neurons (Fig. 4c,d and Extended Data Fig. 10d,e). Removing Side-II from T4/T5 neurons produced a similar graded phenotype affecting dorsal, but not ventral, LPLC2 neurons (Fig. 4e). Thus, the severity of the loss-of-function phenotype correlated with the *beat-VI* expression level across LPLC2 neurons.

That *beat-VI* functions in an instructive manner was supported by the finding that overexpression of *beat-VI* selectively in LPLC2 caused the opposite effect, increasing LoP4 dendritic branch length in ventral, but not dorsal, LPLC2 neurons (Extended Data Fig. 10f–h). This phenotype corresponded to a relatively stronger increase in *beat-VI* mRNA levels across ventral, rather than dorsal, LPLC2 neurons (280% versus 81%, respectively; Extended Data Fig. 4h). These findings indicate that graded Beat-VI–Side-II interactions regulate synaptic connectivity between T4d/T5d axons and LPLC2 dendrites. As the dendrites of ventral LPLC2 neurons are unaffected in *beat-VI* and *side-II* removal, these dendrites must be regulated by a Beat-VI-independent mechanism or by such a mechanism acting in a redundant manner with Beat-VI and Side-II.

**Fig. 5:**
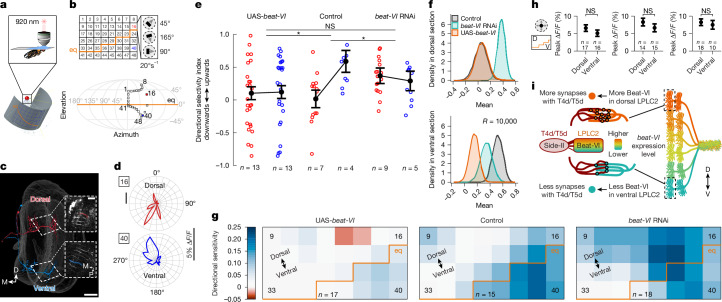
A gradient of Beat-VI–Side-II interactions controls downwards motion perception in LPLC2. **a**, Schematic of the fly eye relative to the display for visual stimulation during calcium imaging. Schematic adapted from ref. ^53^, eLife Sciences Publications. **b**, Display positions probing LPLC2 receptive field with dark edges moving in 24 orientations (top). The orange line indicates the eye equator (eq) projected onto the display. Mollweide projection of the outermost positions (black boxes) relative to the eye is also shown (bottom). **c**, Connectomic reconstructions^16^ of putative LPLC2 neurons at positions 16 (bodyID_28871) and 40 (bodyID_30207). The insets show neurites imaged; single-cell ROIs are overlaid. Scale bars, 25 μm. P, posterior. **d**, Polar plots of peak responses to moving dark edges in dorsal (red) and ventral (blue) LPLC2 neurons (representative fly). **e**, Directional sensitivity index (DSI) for dorsal (red) and ventral (blue) LPLC2 neurons in control, *beat-VI*RNAi and UAS-*beat-VI* flies. The circles indicate individual recordings, and the lines denote position–genotype interactions. The error bars indicate mean ± s.e.m. A two-way rANOVA revealed a genotype × position interaction (*χ*^2^ = 9.80, *P* = 0.0074). *P = 0.0192 (UAS-*beat-VI* versus control), *P = 0.0173 (*beat-VI*RNAi versus control) and NS *P* = 1.0000 (UAS-*beat-VI* versus *beat-VI* RNAi). Bonferroni-adjusted pairwise contrasts. **f**, Bootstrap of DSI mean in dorsal (16; top) and ventral (40; bottom) regions for control, *beat-VI* RNAi and UAS-*beat-VI* flies. **g**, Filtered heatmap of DSI for all tested positions in control, *beat-VI* RNAi and UAS-*beat-VI* flies. **h**, Average peak responses to looming stimuli above (dorsal) or below (ventral) the eye’s equator in UAS-*beat-VI* (left), control (middle) and *beat-VI* RNAi (right) flies. The error bars denote mean ± s.e.m. A two-way rANOVA revealed a main effect of position (*χ*^2^ = 75.75, *P* < 0.0001). Bonferroni-adjusted pairwise *t*-tests for post-hoc comparisons. NS *P* = 1.0000 for all comparisons. **i**, The model shows that a DV gradient of Beat-VI–Side-II interactions determines synapse number between T4d/T5d and LPLC2 neurons. *n*, biologically independent animals (multiple trials per animal; **e**,**g**,**h**). Data are from single experiments. Source data

**Extended Data Fig. 11:**
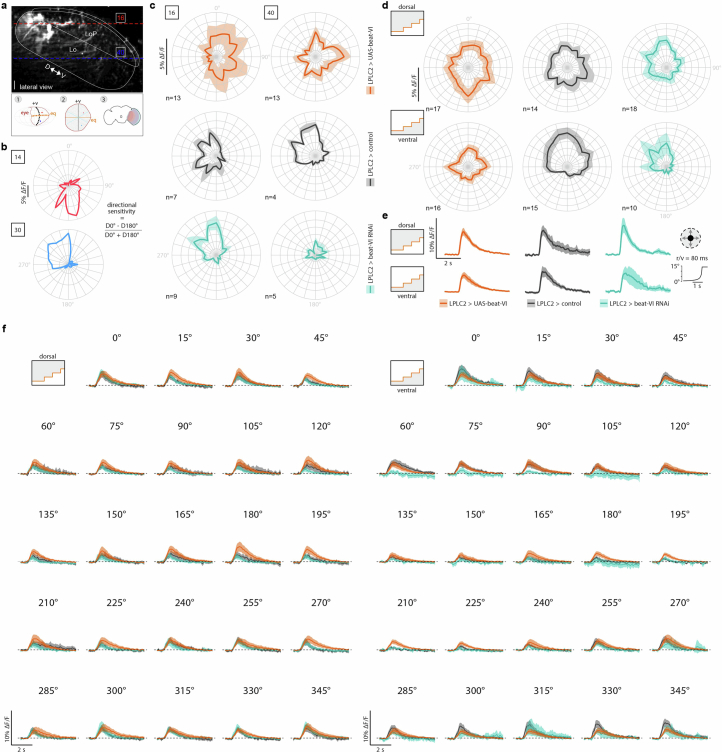
(related to Fig. 5). Beat-VI::Side-II molecular gradient regulates T4d/T5d-LPLC2 synaptic gradient. **a**, 3D reconstruction of LPLC2 neurons from a Z-stack taken using the two-photon microscope in a head-fixed fly (lateral view, scale bar: 10 μm). Dashed lines highlight the two Z-planes where LPLC2 dendrites responded to dark looming at positions 16 (red) and 40 (blue) on the LED display (top). White solid lines approximately define the reference neuropils. Schematic of the procedure used to identify the putative neurons in the fly connectome stimulated by two representative grid positions (bottom, see Methods). D, dorsal; V, ventral. **b**, Polar plots of the peak responses to moving dark edges in dorsal (14) and ventral (30) regions in two representative flies (color-coded by position). **c**, Polar plots of the average peak responses to moving dark edges in dorsal (16) and ventral (40) regions for control, LPLC2>*beat-VI* RNAi, and LPLC2 > UAS-*beat-VI* flies. Error band, ± s.e.m. **d**, Polar plots of the average peak responses to moving dark edges in aggregated dorsal (above the equator) and ventral (below the equator) regions for control, LPLC2>*beat-VI* RNAi, and LPLC2 > UAS-*beat-VI* flies. Error band, ± s.e.m. **e**, Average calcium transient in response to a dark looming in dorsal (above the equator) and ventral (below the equator) regions for control, LPLC2>*beat-VI* RNAi, and LPLC2 > UAS-*beat-VI* flies. As the looming response is the non-linear sum of the response of dendrites in all for LoP layers, the small effects on the response to motion in LoP4 do not alter the measurement of the looming response in this assay. Error band, s.e.m. **f**, Average calcium transients in response to dark edges moving in 24 (from 0° to 345°) different orientations for control, LPLC2>*beat-VI* RNAi, and LPLC2 > UAS-*beat-VI* flies. Dorsal (left) and ventral (right) regions. Error band, ± s.e.m. Panels **c-d**: n values represent biologically independent animals (multiple trials per animal). Source data

To determine whether loss of Beat-VI in LPLC2 has functional consequences, we assessed the response of LPLC2 neurons in head-fixed animals to small dark bars moving in different directions across a white background using calcium imaging (Fig. 5a–c and Extended Data Fig. 11a). Wild-type dorsal and ventral LPLC2 neurons exhibited different responses to these directional stimuli (Fig. 5d–g and Extended Data Fig. 11b–d,f). Ventral LPLC2 neurons showed little to no response to downwards motion, as would be expected if they received little input from downwards sensing T4d/T5d neurons in LoP4 (Figs. 4b and 5d–g and Extended Data Fig. 11b,c,f). By contrast, dorsal LPLC2 neurons were more sensitive to motion in both upwards and downwards directions (Fig 5d–g and Extended Data Fig. 11b,c,f). In response to *beat-VI* knockdown, dorsal LPLC2 neuron responses became biased for upwards motion similar to wild-type ventral neurons. This is consistent with the reduction of downwards-selective T4d/T5d input (that is, loss of LoP4 dendritic branches; Fig. 4c). However, *beat-VI* knockdown had little effect on ventral LPLC2 neurons, which remained biased for upwards motion (Fig. 5d–g and Extended Data Fig. 11c,f). Uniform overexpression of *beat-VI* in LPLC2 neurons superimposed on the normal *beat-VI* expression pattern caused the opposite effect. Dorsal neurons remained unaffected, whereas ventral neurons gained downwards motion sensitivity, making bidirectionally responsive-like wild-type dorsal neurons (Fig. 5d–g and Extended Data Fig. 11d,f). Thus, both *beat-VI* knockdown and overexpression disrupted the normal directional tuning biases across LPLC2 neurons (Fig. 5g,h and Extended Data Fig. 11e).

In summary, Beat-VI and Side-II act as a ligand–receptor pair to control synaptic connectivity between T4d/T5d and LPLC2 neurons. Our data support a model in which the dorsoventral gradient of Beat-VI–Side-II interactions contributes to the formation of the dorsoventral gradient of synaptic weights between T4d/T5d and LPLC2. This results in a decreased response to downwards motion by LPLC2 neurons with ventral receptive fields (Fig. 5i).

## Discussion

In this study, we identified the molecular origins of two parallel dorsoventral synaptic gradients in the axons and dendrites of looming-detecting LPLC2 neurons. These synaptic gradients translate retinotopic information from the visual field of the fly into specific motor outputs. We showed that a gradient of Beat-VI through interactions with its binding partner Side-II regulates graded synaptic inputs from T4d/T5d axons onto LPLC2 dendrites. This regulates differential integration of the downwards motion component of the looming stimulus across different regions of the eye. Similarly, a gradient of Dpr13 working through its binding partner DIP-ε shapes graded synaptic outputs of LPLC2 onto the GF. This biases the escape towards faster takeoffs in response to looming stimuli from the dorsal visual field.

These synaptic gradients, however, are distinct from each other. The dendritic synaptic gradient follows a retinotopic match between populations of presynaptic (T4d/T5d) and postsynaptic LPLC2 neurons (that is, many to many with retinotopy). By contrast, the axonal synaptic gradient follows a many-to-one pattern, in which multiple LPLC2 axons form different numbers of synapses onto a single GF neuron, independent of retinotopy. Despite these differences, both synaptic gradients rely on varying levels of recognition proteins across LPLC2 neurons, which establish synapse numbers that ultimately shape behaviour.

The relationship between the gradients of *beat-VI* and *dpr13* expression and the number of synaptic inputs to and outputs from LPLC2 was also explored. In *beat-VI* loss-of-function and gain-of-function experiments, the severity of functional effects and the extent of branching in LoP4 correlated with *beat-VI* expression levels. It remains unclear whether this phenotype arises from a failure to extend dendrites or to stabilize them. We propose that Beat-VI and Side-II promote adhesion between T4d/T5d axons and LPLC2 dendrites. This protein interaction could influence synapse number either indirectly, by increasing the area of contact between T4d/T5d axons and LPLC2 dendrites, or directly, by controlling synapse formation.

Similarly, Dpr13–DIP-ε interactions are required for LPLC2–GF synaptic connectivity. Loss of DIP-ε reduced the overlap between LPLC2 axons and GF dendrites, leading to significant defects in GF responses to looming. Analysing the loss of Dpr13 function is complicated by the expression of four additional Dpr paralogues in LPLC2, which bind to DIP-ε with similar affinities^37^. By ectopically expressing *DIP-ε* in LPLC2, we inhibited all DIP-ε-interacting Dprs in LPLC2. This mimicked the DIP-ε loss-of-function phenotype in the GF and provided additional support for the interactions between DIP-ε in the GF dendrites with its cognate Dprs in LPLC2 axons. We propose that multiple Dpr paralogues establish baseline LPLC2–GF connectivity, whereas Dpr13 specifically defines the synaptic gradient. In support of this, both gain and loss of Dpr13 function eliminated the graded behavioural response to dorsal versus ventral looming stimuli. Thus, Dpr13–DIP-ε interactions precisely regulate synapse number, forming a behaviourally relevant synaptic gradient. Future studies will explore the mechanistic basis of this regulation.

The Dpr13–DIP-ε gradient differs from Eph–Ephrin gradients in vertebrate retinotectal maps^44^. Although both relay visual-spatial information, they use different strategies. Ephrins and Ephs form topographical maps by maintaining retinotopy, ensuring the spatial relationship between retinal ganglion cells in the retina and the location of their axon terminals in the midbrain^45^. By contrast, the Dpr13–DIP-ε gradient translates retinal positional information into synapse numbers in the brain independent of anatomical mapping.

As most VPNs form synaptic gradients^5^, differential expression of recognitions molecules may be a common mechanism for synapse number regulation. In mammals, altering levels of SynCAM1, another IgSF molecule, also affects synapse number in the hippocampus^46^. Thus, cell recognition molecules not only match synaptic partners^32,47^ but their expression levels may also influence the number of synapses between matched neurons with functional and behavioural consequences. This variation holds enormous potential for fine-tuning behavioural responses driven by neural circuits.

Our work reveals continuous molecular heterogeneity within VPN cell types. This differs from discrete^27,30^ or stochastic^48^ variations in gene expression within neuronal types previously described in *Drosophila*. Beyond recognition molecules, we identified many graded genes in VPNs, suggesting that molecular gradients may regulate broader cellular functions. Cell types in the mammalian cortex also exhibit spatially patterned transcriptomic continuity^7,49,50^. Thus, within-cell-type molecular variation could be a common mechanism for generating neuronal diversity which, in turn, contributes to synaptic specificity.

In conclusion, we have described a molecular strategy regulating neural circuit assembly. This became possible by combining detailed electron microscopy connectomics^16,21–23^, single-cell genomics, genetics, physiology, biochemistry and behaviour. We anticipate that merging these approaches to study other circuits in the fly and mammalian brain^51,52^ will uncover new molecular principles underlying wiring specificity.

## Methods

### Experimental model details

Flies were reared under standard conditions at 25 °C and 50% humidity with a 12-h light–12-h dark cycle on a standard cornmeal fly food. Supplementary Table 1 provides detailed descriptions of fly genotypes used in each experiment and origins of transgenic stocks. For developmental experiments, white pre-pupae (0 h APF) were collected and incubated at 25 °C for the indicated number of hours.

### scRNA-seq experiment

Virgin female flies carrying LPLC2, LPLC1 or LC4 split-GAL4 driver lines were crossed to male flies expressing a nuclear GFP reporter and carrying unique 3rd chromosomes from the isogenic wild-type strains with known genotypes (*Drosophila* Reference Genetic Panel (DGRP)^29^). Each experimental condition (cell type and time point) was crossed to six unique DGRP strains (see Supplementary Table 1 and Source Data for details; the LC4 driver was crossed into four DGRP strains for 72 and 96 h APF time points). Each of these crosses was considered independent biological replicates. F1 generation animals were collected at 0 h APF and incubated for either 48, 72 or 96 h at 25 °C. To introduce a control for developmental age, we split six DGRPs (for each cell type and time point) into equal ‘early’ and ‘late’ groups (Fig. 1h; see Source Data for a detailed breakdown). Animals from early DGRPs were continuously staged and collected within the 2-h time window, after which we continuously staged and collected animals from late DGRPs within the next 2-h time window. This ensured that by the time brains were dissected (after 48, 72 or 96 h), animals from the early group were on average 2 h older than their late counterparts. Brain dissociation was performed as previously described^27^. Single-cell suspensions were used to generate scRNA-seq libraries using the 10X Genomics Chromium Next GEM Single Cell 3′ kit (v3.1) following the manufacturer’s protocol. Each sample (corresponding to a single time point) was loaded to a single lane of 10X Chromium. The libraries were sequenced using NovaSeq 6000 platform, with 100 bp + 100 bp reads for the 48-h sample and 28 bp + 91 bp reads for the 72-h and 96-h samples. Library preparation and sequencing were performed by the Technology Center for Genomics and Bioinformatics at UCLA.

### scRNA-seq data processing and analysis

Raw scRNA-seq reads were processed using Cell Ranger (10X Genomics, v7.1.0). The reference genome and gene annotations were downloaded from FlyBase^54^ (release 6.29). Each time point was processed separately. Six biological replicates were tagged with a unique wild-type chromosome, and demultiplexed based on a unique wild-type chromosome using demuxlet^28^ (v2; https://github.com/statgen/popscle). Demultiplexing was performed using single-nucleotide polymorphisms (SNPs) from seven DGRP strains used in experiments (see Supplementary Table 1 for the full list of genotypes and Source Data for the list of DGRP strains used for each genotype and time point) and three additional DGRP strains as negative controls (line_129, line_427 and line_712). SNPs were filtered using the following criteria: (1) only biallelic SNPs on the 3rd chromosome without missing data (called in all 10 strains); (2) non-reference allele only in 1 of 10 strains. We quantified allelic counts for filtered SNPs using samtools mpileup^55^ (v1.10). SNPs with a minimum total coverage of 10 in all three samples (time points) and a maximum non-reference allele frequency of 0.25 were kept for downstream analysis (34,655 SNPs). Only a few cells (0.1–0.2%) were erroneously assigned to negative controls; 5–15% of cells were classified as ‘doublets’ and ‘ambiguous’ (mostly cells with low transcript coverage). We removed cells with less than 10,000 or more than 50,000 transcripts per cell (and more than 10% of mitochondrial transcripts). The final dataset included 2,595 cells for 48 h APF, 2,369 cells for 72 h APF and 1,039 cells for 96 h APF.

The scRNA-seq analysis was performed using Seurat^56^ package (v5.0.1). The analysis was performed separately for each time point using the standard Seurat workflow: raw transcript counts were normalized, 1,000 highly variable genes were scaled and used for PCA, first 5 PCs were used for clustering (resolution of 0.05) and to calculate tSNE projections. We used tSNE projection only for the summary visualization of the dataset. Clusters were annotated based on known marker genes^27^ (Fig. 1i,j and Extended Data Fig. 2a,b). Most of the cells corresponded to LPLC2, LPLC1 and LC4 neurons. To explore further heterogeneity within VPN type, we subsetted each cell type at each time point, identified 500 highly variable genes and repeated PCA. We plotted known biological and technical covariates along each analysed PC (Fig. 1l and Extended Data Figs. 2 and 3), including developmental age, DGRP genotype, sex and coverage (that is, transcripts per cell). PC1s in LPLC2 and LPLC1 did not correlate with any of these covariates and were driven by similar sets of genes (Fig. 1l,m and Extended Data Fig. 2c,d). Further in vivo validations using orthogonal approaches confirmed that these PCs captured true molecular heterogeneity within each of these cell types (Fig. 2).

### Behavioural experiments

#### High-throughput takeoff assay

A high-throughput takeoff assay was performed with the FlyPEZ system^25^, which allows for the near-automated collection of fly behaviours in response to visual stimulation in large sample sizes. FlyPEZ experiments were performed as previously described^25^. A single stimulus was presented per fly. All behavioural experiments were performed 4 h before incubator lights were switched off, which coincides with the activity peak of the flies in the afternoon light cycle.

#### Visual stimulation for behavioural assay

A 7-inch-diameter back-projection-coated dome was placed centred over the glass platform to present visual stimulation. Specifically, dark looming disks that approach the fly from azimuth of 0° (front looms) or 90° (side looms), at elevations of −30°, 0°, 23°, 45° or 77° in fly coordinates, were used. Looming stimuli were generated using the same equation as described for calcium imaging experiments (see below). All looming stimuli have *l*/*v* = 40 ms. Experiments in Fig. 1e and Extended Data Fig. 1a–c show trials that were performed in the past (from 2014 to 2024) using control flies shown looming disks with a starting size ranging from 1–30° expanding to either 45°, 90° or 180°. Experiments in Fig. 3 include trials with looming disks expanding from 10° to 180° only.

#### Behavioural data analysis

To quantify the duration of the takeoff sequence, videos were manually annotated to identify the start of the sequence (the first frame of wing rising) and the end of the sequence (the last frame that shows T2 legs in contact with the platform). Takeoff sequence durations between 0 ms and 7 ms were considered short-mode takeoffs, and takeoff sequence durations longer than 7 ms were considered long-mode takeoffs, as previously described^20^. The total takeoff percentage was calculated by the number of takeoffs divided by the total number of trials. Short-mode takeoff percentage was calculated by the number of short-mode takeoffs divided by the total number of takeoffs. For experiments in Fig. 1e and Extended Data Fig. 1a–c, takeoff sequence duration longer than 50 ms was eliminated as outliers. All takeoff sequence durations were less than 50 ms for experiments in Fig. 3.

#### Statistical analysis

Statistical comparison of the percentages of short-mode takeoffs was performed with the Chi-squared test, with post-hoc Bonferroni correction for multiple comparisons. Statistical comparison of takeoff sequence distributions between two samples was performed with the Mann–Whitney *U*-test. Statistical comparison of takeoff sequence distributions between more than two samples was performed with the Kruskal–Wallis test, with post-hoc Dunn correction for multiple comparisons. Analysis and plotting were conducted with custom scripts in MATLAB 2022b, and Scipy 1.13.0 and Seaborn 0.13.2 in Python 3.

### Electrophysiological experiments

#### Electrophysiological recordings

In vivo whole-cell, current-clamp electrophysiology was performed on behaving, tethered flies as previously described^57^.

#### Visual stimulation for electrophysiology

Visual stimuli were back-projected onto a 4.5-inch diameter mylar cylindrical screen covering 180° in azimuth via two DLP projectors (Texas Instruments Lightcrafter 4500) as previously described^57^.

For electrophysiology experiments in Fig. 1, visual stimuli were back-projected at 360 Hz onto a 4-inch diameterdome at 768 × 768 resolution as previously described^5^. Looming visual stimuli were generated using Psychtoolbox as previously mentioned. To maximize GF responses, a column of three black looming disks was displayed on a white background on the experimentally accessible visual field of the fly from elevation of −25° to 25°. The looming disks expand from 0° to 30° at a constant velocity of 500° s^−1^. Looming stimuli from different elevations were shown in randomized order for five times per animal, with a 15-s inter-stimulus interval. The baseline region of each trial corresponded to the 2-s time window before the onset of the looming stimulus, and the response region was the 150-ms period after the onset of the stimulus. To estimate the magnitude of depolarization in response to looming stimuli, membrane potentials were averaged across individual trials, and the peak response (mV) and area (ms × mV) relative to the baseline were calculated in the 150-ms response region using custom MATLAB scripts. Statistical comparison of the looming responses in the GF across elevations was performed with repeated-measures one-way ANOVA test, with post-hoc Sidak correction for multiple comparisons.

### Two-photon calcium imaging experiments

#### Imaging setup

Calcium imaging was performed with a VIVO Multiphoton Open (Intelligent Imaging Innovation, Inc.) system based on a moveable objective microscope (Sutter Instruments). The excitation of the sample was delivered by a Ti:Sapphire laser (Chameleon Vision I, Coherent) tuned to 920 nm with power ranging from 15 to 30 mW (depending on imaging depth). A dual axis mirror galvanometer was used for *x*–*y* laser scanning (RGG scanbox, Sutter Instrument). We imaged with a ×20 water-immersion objective (W Plan-Apochromat ×20/1.0 DIC, Zeiss) and a band-pass filter (Semrock 525/40 nm) was placed in front of the photomultiplier tube (H11706P-40, Hamamatsu) to reduce the blue light from the visual display. Microscope and data acquisition were controlled by Slidebook 2024 (Intelligent Imaging Innovation, Inc.). Single plane images were sampled at 9 Hz with a spatial resolution of approximately 180 × 180 pixels (95.7 × 95.7 μm, pixel size ≅ 0.53 μm and dwell time ≅ 2 μs). Images and external visual stimuli were synchronized a posteriori using frame capture markers (TTL pulses from Slidebook 2024) and stimulus events (analogue outputs from the visual display) sampled with a data acquisition device (USB-6229, National Instruments) at 10 kHz.

#### Fly tethering and preparation for imaging

Flies were prepared and head-fixed to a custom objective stage fly holder as previously described^53^. The cuticle above the right optic lobe was removed and the brain bathed in isotonic saline. The holder with the tethered fly was placed under the objective at the centre of the visual display in the horizontal plane. GCaMP7f responses of dendritic branches from individual LPLC2 neurons were recorded from a posterior view. The fly head was pitched forwards, pointing down at the visual display so that the equator of the fly eye held a pitch angle of approximately 60° relative to the imaging plane. For each fly, we identified the most dorsocaudal dendritic arbors in the LoP and then moved the focal plane approximately 10 μm below them to start mapping the receptive field centres of dorsal LPLC2 neurons, or moved approximately 50 μm to probe ventral LPLC2 receptive fields, similarly to previous calcium imaging experiments in LPLC2 (ref. ^6^). Random steps (±5 μm) between these two bracketed *Z*-planes were used to probe the receptive field centres of dorsoventrally intermediate LPLC2 neurons. Unstable recordings or recordings from preparations that did not respond during the receptive field scanning trials were not included in the dataset.

#### Visual stimuli for imaging

A visual display composed of 48 8 × 8 dot matrix LED panels arranged in a semi-cylinder^58^ was used for visual stimulation as previously described^53^. Four layers of filter (071, LEE Filters) were placed over the display to reduce its light intensity. To compensate for the angle of the eye’s equator and optimize the extension of the surrounding visual context, the display was tilted forwards at an angle of 30° from the horizontal plane. Visual presentation was confined to the right half of the visual field, ipsilateral to the recording site. Visual stimuli were generated and controlled using custom-written MATLAB (MathWorks) scripts that communicated to the display through the microcontroller serial port. Looming stimuli simulated an object approaching the fly at a constant velocity, equivalent to twice the inverse tangent of the ratio between the half-size and the approach speed of the object (see description of electrophysiological experiments). The display background was set to 70% maximum intensity, whereas foreground objects (looming or moving bars) were set to 0%. The set of visual stimuli was presented in random block design and repeated two times. Each visual stimulation lasted 4 s and was composed by 0.5 s of uniform background, and 0.5 s of visual motion followed by 3 s of static pattern. Each trial was followed by 3 s of rest in which flies faced the visual background.

#### Receptive field centre and directional tuning

We imaged from the unbranched neurite that connects an LPLC2 dendrites in the LoP to their dendrites in the lobula. Neurites in this location were previously shown to have weak responses to a small bar moving in each of four cardinal directions (that is, stimuli exciting LPLC2 dendrite branches in a single LoP layer) and a much larger response to looming (that is, stimuli exciting LPLC2 dendritic branches in all four LoP layers simultaneously)^6^. We identified an active neurite from a single neuron in the multiphoton field of view (Extended Data Fig. 11a). The receptive field centre of that neurite was identified in real-time and subsequently scanned for directional sensitivity. We developed a custom GUI in MATLAB, which allowed for real-time modifications to stimulus positions on the visual display. This interface enabled hand-triggered looming stimuli and the visual inspection of GCaMP responses. To identify the receptive field centre of an LPLC2 dendritic branch, we created a rectangular grid of 48 positions across the right half of the visual display.

The positions were spaced every five LEDs in both horizontal and vertical directions, with each LED covering approximately 2.2° on the retina at the eye’s equator. Using the GUI, the experimenter presented a looming stimulus centred at each grid position. The looming stimulus simulated a circular object with a 0.5-cm radius, starting from a distance of 50 cm and travelling at 62.5 cm s^−1^. This caused the object to expand from 0.6° to 14° with a loom velocity (*l*/*v*) of 8 ms. If a response was visually detected, the surrounding grid positions were probed next. The position with the highest peak response was taken as the receptive field centre for the subsequent directional tuning experiment. We tested directional selectivity by moving a dark edge outwards from the centre of the receptive field in 24 different directions (Fig. 5b, top, and Extended Data Fig. 11). The edges moved at 20° s^−1^ with orientations ranging from 0° to 345° in 15° increments. Each edge subtended 15° at the eye’s equator and swept 15° orthogonal to its orientation, filling a 15° black square upon completion. In addition, a looming stimulus centred within the receptive field, with the same dynamics as those used for the receptive field scans, was included in this battery of dark moving edges.

#### Mapping LPLC2 position and directional sensitivity index

We sampled neurons along the dorsoventral and anteroposterior axes of the lobula and confirmed their anatomical locations by mapping the receptive field centres onto the fly eye (Fig. 5b, bottom). To identify the putative individual LPLC2 neurons stimulated by the receptive field centre scans, we mapped the horizontal coordinates of their retinal positions onto the 2D retinal ommatidia lattice. We identified specific dorsal and ventral retinal ommatidia and their corresponding columnar LPLC2 in the connectome, verifying the recorded locations of LPLC2 neurons (Fig. 5c and Extended Data Fig. 11a). Coordinates were calculated using a 3D reconstruction of the fly head, holder and visual display in AutoCAD (Autodesk). We estimated the fly ommatidia with overlapping horizontal coordinates through the following steps: (1) identified the locations of the ommatidia pointing to positions 16 and 40 based on a Mollweide projection of 3D ommatidia directions from a microCT scan^59^; (2) mapped these ommatidia locations onto identified visual columns of the male optic lobe connectome^16^; (3) used T4 neurons included in these visual columns to identify downstream LPLC2 neurons.

For each recording, the direction sensitivity index (DSI) was computed as follows:

$${\rm{DSI}}=({R}_{{\rm{up}}}-{R}_{{\rm{down}}})/({R}_{{\rm{up}}}+{R}_{{\rm{down}}})$$

where ${R}_{{\rm{up}}}$ is the peak response to an upwards moving edge (0° direction) and ${R}_{{\rm{down}}}$ is the peak response to a downwards moving edge (180° direction). The index ranged from −1 to 1, with negative values indicating downwards sensitivity and positive values indicating upwards sensitivity. The heatmap of the DSI for the tested positions was smoothed with a Gaussian filter (σ = 1).

#### Imaging data analysis

Images exported from Slidebook 2024 were processed following established protocols^53^. We used a custom MATLAB toolbox developed by B. J. Hardcastle (available at https://github.com/bjhardcastle/SlidebookObj) to correct for motion artefacts in the *x*–*y* plane and to delineate regions of interest (ROIs) around individual LPLC2 neurites within the dendritic tree. For each recording, a time series was generated by calculating the mean fluorescence intensity of pixels within the ROI (_*Ft*_) in each frame. These mean values were then normalized to a baseline value using the formula:

$$\Delta F/F=({F}_{t}-{F}_{0})/{F}_{0}$$

where ${F}_{0}$ is the mean of _*Ft*_ during the 0.5 s preceding stimulus onset. This approach ensures accurate correction for motion artefacts and reliable quantification of fluorescence intensity changes in LPLC2 neurites.

#### Statistical analysis of calcium imaging data

The time series for each ROI were then exported from MATLAB and imported in RStudio by using the R package ‘R. matlab’^60^. Custom R scripts were then written for data plotting and statistical analyses. Given the repeated sampling and unbalanced sample sizes between groups and conditions, we used linear mixed effects models to fit the DSI values. This method maintains statistical power by avoiding averaging procedures and provides more accurate estimates of model parameters, including both fixed and random effects. The fixed effects were defined by the interaction between genotype (control, *beat-VI* RNAi or UAS-*beat-VI*) and condition (dorsal or ventral), whereas the random effects were attributed to individual flies^61,62^. We modelled the data using the R package ‘lme4’^63^ assuming residuals followed a Gaussian distribution. ANOVA was then run for the model by using the R package ‘car’^64^. Pairwise post-hoc comparisons of the fixed effects were conducted using *t*-tests with Bonferroni adjustments, implemented through the R package ‘emmeans’^65^. With the same package, we calculated the Cohen’s *d* effect sizes as the pairwise difference between model estimates divided by the standard deviation of the data (Supplementary Table 2). In addition, to estimate the mean DSI differences across groups and conditions without assuming a specific distribution, we performed standard bootstrap simulations with 10,000 replicates using the R package ‘boot’^66,67^. Dot plots were generated with the R package ‘ggplot2’^68^. Smoothed heatmaps were generated with the R package ‘spatstat’^69^.

### Generation of transgenic flies

#### UAS-*DIP-ε*, UAS-*dpr13* and UAS-*beat-VI* transgenic flies

The coding sequences of *DIP-ε*, *dpr13* (isoform RB) and *beat-VI* were cloned into a modified pJFRC5 vector (Addgene: 5×UAS-IVS-mCD8::GFP, plasmid #26218) by replacing the mCD8::GFP coding sequence. Cloning strategies were designed using SnapGene 4.1.9 (GSL Biotech). Synthesis and cloning were carried out by Genewiz, Inc. Plasmids and sequences are available on request. Flies were generated by injecting the plasmid into embryos for recombination into attP1 sites (BDSC #8621) by BestGene, Inc.

#### *DIP-ε* and *dpr13* null alleles

The *DIP-ε*^*null*^ allele was generated as previously described^70^. In brief, two single guide RNAs (sgRNAs) were used to generate a frameshift deletion in the *DIP-ε* coding sequence. High-score spacer sequences were chosen using the SSC tool^71^. Each sgRNA was cloned into pU6-2-sgRNA-short (Addgene #41700) plasmid and two plasmids were co-injected into the vas-Cas9 line (BDSC #51324) by Bestgene, Inc. Injected larvae were crossed with balancer lines and PCR screened in F1 for single flies carrying the deletion. A mutant stock was established from this single F1.

The sgRNA target sequences used for *DIP-ε*^*null*^ allele generation were GCTGTTCTGTGGTCATACGATAGC and CTTCAATCGATTGACGGTGGAGC.

The *dpr13*^*null*^ allele was similarly generated. sgRNA sequences were identified with an efficiency score above 5, as defined by the CRISPR Efficiency Predictor (https://www.flyrnai.org/evaluateCrispr/). The sgRNA sequence oligos were synthesized (Integrated DNA Technologies) and cloned into the pU6b-sgRNA-short vector^72^ to generate a large approximately 30-kb deletion spanning most of the *dpr13* genomic region. All pU6 vectors generated were verified by Sanger sequencing. Two plasmids were co-injected into the vas-Cas9 line (BDSC #51323) in Bestgene, Inc. Injected larvae were crossed with balancer lines and PCR screened in F1 for single flies carrying the deletion. A mutant stock lacking the entire coding sequence of *dpr13* was established from this single F1.

The sgRNA target sequences used for *dpr13*^*null*^ allele generation were CGATATAATCCACTTGATGC and ACGTAGCAGCTCCAGGATGT.

Detailed protocols are available on request.

### Immunohistochemistry and DPX mounting

All protocols in immunohistochemistry and DPX mounting were performed exactly as described in our previous study^5^.

### Antibody information

#### Primary antibodies and dilutions

Chicken anti-GFP (1:1,000; Abcam #ab13970, RRID: AB_300798), rabbit anti-dsRed (1:200; Clontech #632496, RRID: AB_10013483), mouse anti-Bruchpilot (1:20; DSHB Nc82, RRID: AB_2314866), chicken anti-V5 (1:200; Fortis Life Sciences #A190-118A, RRID: AB_66741), mouse anti-V5 (1:500; Abcam #ab27671, RRID: AB_471093), rabbit anti-HA (1:200; 3724, Cell Signaling Technology, RRID: AB_1549585), rabbit anti-FLAG (1:200; Abcam #ab205606, RRID: AB_2916341), rat anti-N-cadherin (1:40; DSHB MNCD2, RRID: AB_528119), anti-GFP nanobody (1:200 for expansion microscopy and 1:500 for confocal microscopy; N0304-At488-L, NanoTag Biotechnologies, RRID: AB_2744629) and rat anti-HA (1:500 for expansion microscopy; 3F10, Roche, RRID: AB_2314622).

#### Secondary antibodies and dilutions

Goat anti-chicken AF488 (1:500; A11039, Invitrogen, RRID: AB_2534096), goat anti-mouse IgG2A (1:500; A21131, Invitrogen, RRID: AB_2535771), goat anti-rabbit AF568 (1:500; A11011, Invitrogen, RRID: AB_143157), goat anti-mouse AF647 (1:500; 115-607-003, Jackson ImmunoResearch, RRID: AB_2338931), goat anti-rat AF647 (1:500; 112-605-167, Jackson ImmunoResearch, RRID: AB_2338404).

### Confocal image acquisition and processing

Immunofluorescence images were acquired using a Zeiss LSM 880 confocal microscope with 488-nm, 561-nm and 633-nm lasers using Zen digital imaging software with a Plan-Apochromat ×63/1.4 oil DIC M27 objective. Serial optical sections were obtained from whole-mount brains with a typical resolution of 1,024 μm × 1,024 μm, and 0.5-μm-thick optical sections. Image stacks were exported to either Fiji 2.0.0-rc-69/1.52k or Imaris 10.1 (Oxford Instruments) for level adjustment, cropping, removal of off-target brain regions and background noise, and 3D volume reconstructions.

### Analysis of neuronal morphology from image stacks

To measure the axo-dendritic overlap between LPLC2 axons and GF dendrites, confocal image stacks of colocalized LPLC2 glomeruli and GF dendrites were imported into Imaris 10.1 for 3D reconstruction using the Surfaces tool to create masks for membranes of presynaptic and postsynaptic neurons from the corresponding channels. A Surfaces detail value of 1 μm was used for both LPLC2 and GF surfaces to ensure accurate reconstruction. Background subtraction was applied with a diameter of the largest sphere that fits into the object set to 1 μm to minimize noise and nonspecific signals. The overlap between the two reconstructed surfaces was then assessed to quantify the spatial relationship between the LPLC2 axons and GF dendrites. A similar approach was used to measure the overlap between Brp puncta and the GF dendrites in STaR experiments (Extended Data Fig. 7), but the number of overlapping reconstructed surfaces was considered regardless of the overlap. To measure LoP4 dendritic branch length in sparsely labelled LPLC2 neurons, corresponding image stacks were imported into Fiji 2.0.0-rc-69/1.52k, rotated so that LoP3 and LoP4 branches were oriented antiparallel, and the distance from the point of bifurcation to the most distal tip of the LoP4 branch was measured along Lop4. Dorsoventral differences in Beat-VI and Dpr13 at the protein level were measured in FIJI 2.0.0-rc-69/1.52k. ROIs corresponding to the cell bodies dorsal or ventral LPLC2 clones were drawn manually. Mean grey value (average pixel intensity) was used as a proxy of the GFP fluorescence intensity and was measured for two ROIs per sample after background subtraction.

### ExLSM and HCR-FISH

Tissue staining, gelation and expansion for ExLSM protocols were adapted from Sanfilippo et al.^73^ with minor changes. After dissection, fixation and permeabilization, brains were stored in RNAse-free 0.5% PBST containing anti-GFP nanobody (N0304-At488-L, NanoTag Biotechnologies) overnight at 4 °C. All samples were subsequently processed using a protein and RNA retention ExM protocol with minor modifications^73,74^ and minor adjustments for the fly brain^34^.

#### HCR-FISH

The HCR-FISH protocol was adapted from Wang et al.^74^ with minor optimizations for the fly brain^34^. Following digestion with proteinase K, gels with embedded brains were washed three times with PBS, transferred into 24-well plates (4624-24, Laguna Scientific) and digested with DNAse diluted in RDD buffer (RNase-Free DNase Set, 79254, Qiagen) to limit DAPI signal to RNA only and facilitate subsequent analysis for 2 h at 37 °C. After three washes in PBS, gels were equilibrated in probe hybridization buffer (Molecular Instruments) for 30 min at 37 °C, and then transferred to new 24-well plates containing custom-designed probes (Molecular Instruments) diluted in pre-warmed probe hybridization buffer (1 μl of 1 μM stock probe solution per 200 μl of buffer) and left shaking overnight at 37 °C. The following day, gels were washed four times with pre-warmed probe wash buffer (Molecular Instruments) for 20 min at 37 °C, then washed twice for 5 min with SSCT buffer (SSC with 0.05% Triton X-100; AM9763, Thermo Fisher) at room temperature and transferred to new 24-well plates with HCR amplification buffer (Molecular Instruments) for equilibration. Hairpins (HCR Amplifiers, Molecular Instruments) conjugated with AF546 or SeTau647 dyes were diluted in amplification buffer (2 μl of each hairpin per 100 μl of buffer), heat-activated in a thermal cycler (90 s at 95 °C), removed and kept for 30 min at room temperature in the dark. After 30 min, the hairpins were added to the 24-well plates with gels (300 μl per well) and incubated with shaking at room temperature in the dark for 3 h. The hairpin solution was then removed, and the gels were washed four times with SSCT and two times with SSC for 10 min at room temperature in the dark. Gels were subsequently stored at 4 °C in SSC until final expansion.

#### Sample mounting

Samples were expanded to approximately three time in 0.5× PBS containing 1:1,000 SYTO-DAPI (S11352, Thermo Fisher) at room temperature for 2 h before mounting onto PLL-coated coverslips (see description for DPX mounting above). The coverslips were then bonded with Bondic UV-curing adhesive (Bondic starter kit, Bondic) onto a custom-fabricated sample holder (Janelia Tech ID 2021-021) to be suspended vertically in the imaging chamber. Mounted samples were imaged in 0.5× PBS with 1:10,000 SYTO-DAPI after a minimum of 1 h of equilibration in the imaging chamber. Unexpanded gels were stored at 4 °C in 1X PBS + 0.02% sodium azide (S8032, Sigma-Aldrich) for up to 14 days before final expansion and imaging.

#### Light-sheet microscopy

Images were acquired on a Zeiss LS7 microscope equipped with 405-nm, 488-nm, 561-nm and 638-nm lasers. Illumination optics with a ×10/0.2 NA were used for excitation (400900-9020-000, Zeiss). Detection was performed using a W Plan-Apochromat ×20/1.0 DIC M27 water immersion objective (421452-9700-000, Zeiss). The LS7 optical zoom was set to 2.5×, resulting in a total magnification of ×50. DAPI and Alexa Fluor 546 dyes were simultaneously excited by the 405-nm and 561-nm laser lines, and emission light was separated by a dichroic mirror SBS LP 510 with emission filters BP 420-470 (404900-9312-000, Zeiss) and a modified BP 527/23 (ET672/23m, Chroma). Similarly, Alexa Fluor 488 and SeTau647 dyes were simultaneously excited via 488 nm and 638 nm, and the emission was split through a dichroic SBS LP 560 with emission filters BP 505-545 and LP 660 (404900-9318-000, Zeiss). To eliminate laser transmission, a 405/488/561/640 laser blocking filter (404900-9101-000, Zeiss) was added to the emission path. Images were captured using dual PCO.edge 4.2 detection modules (400100-9060-000, Zeiss) with a 50-ms exposure time. Filter and camera alignment were manually calibrated before each imaging session. Image volumes were acquired at optimal *Z*-step and light-sheet thickness, and the Pivot Scan feature was used to reduce illumination artefacts by sweeping the light sheet in the *xy* plane. The LS7 microscope was operated using ZEN Black 3.1 (v9.3.6.393).

### Analysis of HCR-FISH data from ExLSM image stacks

The full details of our analysis are available in our previous publication^35^. The acquired light-sheet *z*-stacks, stored in CZI format, were imported and pre-processed to remove noise and artefacts generated by the imaging modality. These artefacts included limited channel contrast, variations in contrast across images within a dataset, background noise fluctuations due to both intra-channel variations and inter-channel crosstalk, and localized brightness changes caused by varying fluorophore concentrations within and among stained nuclei. Pre-processing involved the following steps: full-scale contrast stretching to normalize luminosity across different channels, local background removal using a 3D Gaussian filter, a second full-scale contrast stretching to compensate for any contrast loss due to background removal, and a final median filter to eliminate any remaining localized noise. These pre-processed stacks served as the starting point for instance segmentation of the nuclei. First, nuclear centres were identified using a Laplacian-of-Gaussian filter. Then, the imaged volume was subdivided into 3D Voronoi cells, using the detected centres as seeds and Euclidean distance. Each cell contained one nucleus, which was segmented using a threshold obtained by minimizing an energy functional designed to find the optimal surface separating the nucleus from the surrounding cytoplasm. Once nuclei were segmented, the FISH puncta were identified within the associated 3D volume using a Laplacian-of-Gaussian filter, and only the puncta within the nucleus region and its immediate surrounding volume were counted. Pre-processed products, segmented nuclear features, associated FISH puncta, and their features were stored for further analysis. Puncta counts were normalized by the maximum count for each brain. The significance of gene expression relationships inferred from HCR-FISH (Fig. 2) was assessed using both a linear regression model and a multi-level negative binomial generalized linear mixed model, accounting for inter-animal heterogeneity: random effects were attributed to individual animals (Supplementary Table 2). The package was written in Python, is open source and is available for download on GitHub (https://github.com/avaccari/DrosophilaFISH).

### Genetic intersection of cell types and genes

To assess gene expression patterns within VPN cell types, we used a combination of GAL4 and LexA binary expression systems. Expression of a LexAop-FRT-stop-FRT-membrane marker controlled by a cell-type-specific LexA driver remained blocked until the FRT-stop-FRT cassette was excised by Flippase, driven by a gene-specific T2A-GAL4 driver line^75^. In addition, a constitutive membrane marker controlled by the same LexA driver was used to visualize the entire cell population. The list of fly stocks used is available in Supplementary Table 1.

### Sparse labelling of neuronal populations

To visualize single-cell morphology of LPLC2 dendrites in the LoP (Fig. 4) and to perform HCR-FISH analysis (Extended Data Fig. 4), we used MultiColor FlpOut^76^, a genetic tool for sparse labelling of individual cells within a population downstream of a given GAL4 driver. Twenty-four-hour-old pupae were heat shocked in a 37 °C water bath for 10 min to achieve the labelling of one LPLC2 neuron per hemibrain on average.

### Analysis of the Flywire connectome reconstruction

To analyse the FlyWire connectome, we developed an open-source Python package, available on GitHub (https://github.com/avaccari/DrosophilaCon). The primary library used by DrosophilaCon to interface with the FlyWire connectome is fafbseg^23,77^ (v3.0.0). This package enables users to specify the labels of ‘source’ and ‘target’ neurons and generates a connectivity diagram where target neurons are colour-coded based on the total count of synapses with the source neurons. Once the labels are specified, the package queries the latest available annotations to identify all neurons matching (or containing) the labels. The primary sources of annotation used to identify the neurons are: (1) free-form community annotations provided through the neuroglancer user interface (https://github.com/google/neuroglancer); (2) systematic annotations for the entire brain^23^; and (3) systematic cell types for the right optic lobe^78^.

Next, the latest version of the FlyWire production dataset was queried for the adjacency matrix representing the connectivity between each neuron in the source and each neuron in the target on the selected side of the brain. This information was returned as an adjacency table, providing the counts of synapses between each source–target pair. There are two versions of the synapse datasets: one filtered by synaptic cleft and one unfiltered. We used the unfiltered dataset because the filtered version applies a fixed threshold for distance, resulting in reduced synapse counts. The adjacency table was used to evaluate the total synapse counts for each target neuron. These counts were normalized by the maximum count observed across all target neurons. The mesh representation for each identified target neuron was downloaded from the FlyWire dataset, skeletonized for optimized processing and visualized with colour-coding corresponding to the normalized synapse count, allowing for comparison across different source–target pairs.

### Statistics and reproducibility

All statistical analyses were performed using RStudio 1.4.1103, MATLAB 2022b or Prism 9.2.0 (GraphPad). Significance levels were defined as follows: *P < 0.05, **P < 0.01, ***P < 0.001 and ****P < 0.0001 for all figures. Statistical tests were chosen based on data distribution, which was assessed using the Kolmogorov–Smirnov test in R with a *P* value threshold of less than 0.05 for normality. Two groups of normally distributed datasets were tested for statistically significant differences using unpaired *t*-tests with Welch’s correction for non-identical variance. For comparisons involving more than two groups, we used either one-way ANOVA followed by Tukey’s honestly significant difference test for post-hoc pairwise comparisons or the Kruskal–Wallis test followed by Dunn’s multiple comparisons post-hoc test with Bonferroni correction. Binary data were compared using Chi-squared tests. Detailed statistical analyses for behavioural data, HCR-FISH data and neuroanatomical data are described in Supplementary Table 2. All other statistical tests, number of replicates, significance levels and other elements of the statistical analysis (including measure of centre and error bars) have been reported within the corresponding figure legends. No data were excluded from the analysis unless specified in the corresponding Methods section. All neuroanatomical and behavioural measurements were taken from distinct samples (that is, individual brains or hemibrains and individual flies, one takeoff per fly).

### Reporting summary

Further information on research design is available in the Nature Portfolio Reporting Summary linked to this article.

## Online content

Any methods, additional references, Nature Portfolio reporting summaries, source data, extended data, supplementary information, acknowledgements, peer review information; details of author contributions and competing interests; and statements of data and code availability are available at 10.1038/s41586-025-09037-4.

## Supplementary information

Supplementary Table 1 This table lists the genotypes of the fly lines used in each experiment of each figure, specifying the neurons targeted by the genetic manipulation, the source, and, for RNAi stocks, the RNAi construct sequence

Reporting Summary

Supplementary Table 2 This file provides a summary of the statistical tests conducted for each experiment, including exact P-values

## Source data

Source Data Figs. 1, 2, 3, 4, 5 and Source Data Extended Data Figs. 1, 2, 3, 4, 6, 7, 8, 9, 10, 11

## Data Availability

The raw scRNA-seq data and the processed dataset are available at NCBI Gene Expression Omnibus: GSE291561. Raw calcium imaging data are available at: 10.17605/OSF.IO/Z7XFK. Confocal stacks^79,80^ (10.5281/zenodo.14968994 and 10.5281/zenodo.14969126), light-sheet stacks^81^ (10.5281/zenodo.14969478) and raw electrophysiological data^82^ (10.5281/zenodo.14983850) are available on Zenodo. All other datasets generated and analysed during the current study (including FlyPEZ videos) are available from the corresponding authors on request. For further information regarding any resources and reagents, please contact S.L.Z. (lzipursky@mednet.ucla.edu) and G.M.C. (gc3017@columbia.edu). Source data are provided with this paper.

## Code availability

The code for scRNA-seq analysis is available on GitHub (https://github.com/kurmangaliyev-lab). The code for the analysis of FlyWire and hemibrain connectome reconstructions is available on GitHub (https://github.com/avaccari/DrosophilaCon and https://github.com/avaccari/DrosophilaVPNWiring, respectively). The code used to analyse HCR-FISH data is available on GitHub (https://github.com/avaccari/DrosophilaFISH). The code related to calcium imaging experiments is available on the Open Science Framework (https://osf.io/z7xfk/).
