# Supplementary material for: Molecular gradients shape synaptic specificity of a visuomotor transformation
Source: Nature. 2025 Jun 4;644(8076):453–62. doi: 10.1038/s41586-025-09037-4 (PMC12350164; doi:10.1038/s41586-025-09037-4)
Supplement: Supplementary file 2 — Reporting Summary [file 41586_2025_9037_MOESM2_ESM.pdf]

nature portfolio

Corresponding author(s): S. Lawrence Zipursky, Gwyneth M. Card

Last updated by author(s): Mar 4, 2025

Reporting Summary

Nature Portfolio wishes to improve the reproducibility of the work that we publish. This form provides structure for consistency and transparency in reporting. For further information on Nature Portfolio policies, see our Editorial Policies and the Editorial Policy Checklist.

Please do not complete any field with "not applicable" or n/a. Refer to the help text for what text to use if an item is not relevant to your study. For final submission: please carefully check your responses for accuracy; you will not be able to make changes later.

Statistics

For all statistical analyses, confirm that the following items are present in the figure legend, table legend, main text, or Methods section.

n/a	Confirmed	
☐	☒	The exact sample size (*n*) for each experimental group/condition, given as a discrete number and unit of measurement
☐	☒	A statement on whether measurements were taken from distinct samples or whether the same sample was measured repeatedly
☐	☒	The statistical test(s) used AND whether they are one- or two-sided<br>*Only common tests should be described solely by name; describe more complex techniques in the Methods section.*
☐	☒	A description of all covariates tested
☐	☒	A description of any assumptions or corrections, such as tests of normality and adjustment for multiple comparisons
☐	☒	A full description of the statistical parameters including central tendency (e.g. means) or other basic estimates (e.g. regression coefficient) AND variation (e.g. standard deviation) or associated estimates of uncertainty (e.g. confidence intervals)
☐	☒	For null hypothesis testing, the test statistic (e.g. *F*, *t*, *r*) with confidence intervals, effect sizes, degrees of freedom and *P* value noted<br>*Give P values as exact values whenever suitable.*
☒	☐	For Bayesian analysis, information on the choice of priors and Markov chain Monte Carlo settings
☒	☐	For hierarchical and complex designs, identification of the appropriate level for tests and full reporting of outcomes
☐	☒	Estimates of effect sizes (e.g. Cohen's *d*, Pearson's *r*), indicating how they were calculated

Our web collection on statistics for biologists contains articles on many of the points above.

Software and code

Policy information about availability of computer code

Data collection	FlyPEZ system (Williamson et al., 2018) was used to obtain high-speed videos of fly escape in order to quantitatively characterize differences in escape behavior. For visual stimulation, we used DMD projectors running at a refresh rate of 360 Hz, controlled by MATLAB 2022b using the Psychophysics Toolbox. Visual stimulation for electrophysiological recordings was performed using two DLP projectors running at a refresh rate of 240 Hz, controlled by MATLAB 2022b using the Psychophysics Toolbox. Whole-cell recordings were performed in current-clamp mode, and digitized at 20 kHz, acquired using the open-source software Wavesurfer running in MATLAB 2022b. Calcium imaging was performed with a VIVO Multiphoton Open system based on a Movable Objective Microscope. The excitation of the sample was delivered by a Ti:Sapphire laser tuned to 920 nm with power ranging from 1.5 to 30 mW. A visual display composed of 48 8x8 dot matrix LED panels arranged in a semi-cylinder was used for visual stimulation (as described in Frighetto et al., 2023). Immunofluorescence images were acquired using a Zeiss LSM 880 confocal microscope using oil-immersion 63x objective. FISH images were acquired using Zeiss LS7 microscope using a W PlanApochromat 20x water immersion objective. Confocal and light sheet images were acquired using with Zen Black 3.1 digital imaging software. Single-cell RNA-seq libraries were generated using the lOX Genomics Chromium Next GEM Single Cell 3'-kit (v3.1) following the manufacturer's protocol. Libraries were sequenced using one lane of NovaSeq 6000 SP platform (28bp + 91 bp). The library preparation and sequencing were performed by the Technology Center for Genomics and Bioinformatics at UCLA.
Data analysis	For behavioral data analysis, videos were manually annotated to identify the start of the sequence (the first frame of wing-rising) and the end of the sequence (the last frame that shows T2 legs in contact with the platform). Takeoff sequence durations between 0ms to 7ms were considered “short-mode takeoffs,” and takeoff sequence durations longer than 7ms were considered “long-mode takeoffs”. Statistical analysis and plotting were conducted with custom scripts in MATLAB 2022b, and Scipy 1.13.0 and Seaborn 0.13.2 in Python 3 (code is available upon request). Whole-cell recording data were analyzed in MATLAB using custom code (available upon request). Calcium imaging data were processed following established protocols (Frighetto et al., 2023). We used a custom MATLAB toolbox developed by Ben J. Hardcastle (available at https://github.com/bjhardcastle/SlidebookObj) to correct for motion artifacts in the x-y plane and to delineate regions of interest

around individual neurites within the dendritic tree. Data and Code are available at https://doi.org/10.17605/OSF.IO/Z7XFK. Post-processing analyses for calcium imaging data were conducted in MATLAB 2019b. Data plotting and statistical analyses for calcium imaging experiments were performed in RStudio (2023.06.0+421) using custom R (4.3.0) scripts. We used the following R packages: R.matlab (3.7.0), lme4 (1.1.35.1), car (3.1.2), emmeans (1.8.9), boot (1.3.28.1), spatstat (3.0.7), ggplot2 (3.4.4). Confocal image stacks were analyzed in either Imaris 10.1, or in FIJI 2.0.0-rc-69/1.52k. "Hemibrain" EM-connectome data was analyzed as previously described (Dombrovski et al., 2023; available at https://github.com/avaccari/DrosophilaVPNWiring). To analyze the Flywire connectome, we developed an open-source Python package, available at https://github.com/avaccari/DrosophilaCon. FISH data was analyzed using an open-source package we previously developed (Vaccari and Dombrovski, 2023), code available at https://github.com/avaccari/DrosophilaFISH. Raw scRNA-Seq reads were processed using Cell Ranger (10X Genomics, version: 7.1.0). The reference genome and gene annotations were downloaded from FlyBase. Biological replicates were tagged with a unique wild-type chromosome, and demultiplexed based on a unique wild-type chromosome using demuxlet (version 2, https://github.com/statgen/popscle), as described in (Kurmangaliyev et al., 2020). The scRNA-seq analysis was performed in RStudio 2023.12.1 using Seurat (5.0.1) and ggplot2 (3.5.0) packages. Code is available at https://github.com/kurmangaliyev-lab. Supplementary Table 2 provides descriptions of all statistical tests used for the analysis of each experiment. Molecular cloning strategies were designed using SnapGene 4.1.9 (GSL Biotech).

For manuscripts utilizing custom algorithms or software that are central to the research but not yet described in published literature, software must be made available to editors and reviewers. We strongly encourage code deposition in a community repository (e.g. GitHub). See the Nature Portfolio guidelines for submitting code & software for further information.

Data

Policy information about availability of data

All manuscripts must include a data availability statement. This statement should provide the following information, where applicable:

- Accession codes, unique identifiers, or web links for publicly available datasets
- A description of any restrictions on data availability
- For clinical datasets or third party data, please ensure that the statement adheres to our policy

Raw calcium imaging data are available at: https://doi.org/10.17605/OSF.IO/Z7XFK.
Confocal images stacks are available at: https://doi.org/10.5281/zenodo.14968994 and https://doi.org/10.5281/zenodo.14969126
Light sheet image stacks are available at: https://doi.org/10.5281/zenodo.14969478
The raw scRNA-seq data and the processed dataset are available at NCBI GEO: GSE291561
Raw electrophysiological data are available at: https://doi.org/10.5281/zenodo.14983850
Due to the large volume of raw, unprocessed videos from the behavioral experiments (thousands of takeoff recordings), we have not deposited them in a public repository due to storage constraints and practicality. However, these videos are available upon reasonable request from the corresponding authors. Videos featured in Figure 1 and Extended Data Figure 1 can be accessed through previous publications (von Reyn et al., 2014; Williamson et al., 2018).

Human research participants

Policy information about studies involving human research participants and Sex and Gender in Research.

Reporting on sex and gender	N/A
Population characteristics	N/A
Recruitment	N/A
Ethics oversight	N/A

Note that full information on the approval of the study protocol must also be provided in the manuscript.

Field-specific reporting

Please select the one below that is the best fit for your research. If you are not sure, read the appropriate sections before making your selection.

☒ Life sciences ☐ Behavioural & social sciences ☐ Ecological, evolutionary & environmental sciences

Life sciences study design

All studies must disclose on these points even when the disclosure is negative.

Sample size

All sample sizes were chosen based on conventional standards used in our fields, considering previously published results. For calcium imaging, see Klapoetke et al., 2017 (https://doi.org/10.1038/nature24626) – calcium imaging from LPLC2 neuronal dendrites, and Frighetto and Frye, 2023 (https://doi.org/10.7554/eLife.83656). For behavioral experiments, see Dombrovski et al., 2023 (https://doi.org/10.1038/s41586-023-05930-y), Williamson et al., 2018 (https://doi.org/10.1016/j.celrep.2018.10.048) – utilizing FlyPEZ apparatus to evaluate looming-evoked responses. For electrophysiological experiments, see von Reyn et al., 2014 (https://doi.org/10.1038/nn.3741) and von Reyn et al., 2017 (https://doi.org/10.1016/j.neuron.2017.05.036) – whole cell recordings from the Giant Fiber. For anatomical experiments, see Xu et al., 2018 (https://doi.org/10.1016/j.neuron.2017.05.036), Tan et al., 2015 (https://doi.org/10.1016/j.cell.2015.11.021) and Yoo et al. 2023

	(https://doi.org/10.1016/j.cub.2023.08.020) – exploring the expression of DIP/Dpr and Beat/Side cell recognition molecules in the fly visual system
Data exclusions	No data were excluded from the analysis except as noted for the behavior experiments (see Methods, "Behavioral Data Analysis").
Replication	For electrophysiological experiments repeated measurements were taken from a given number of animals (n values are indicated in the corresponding Figure Legends of panels). For calcium imaging experiments, the whole set of visual stimuli (i.e., loom + 24 moving edges) was presented 2 times per position. A variable number of positions were recorded per fly (from 1 to 8). For all other experiments (takeoff behavior, neuroanatomy, HCR-FISH), results were replicated in different individual flies/brains across each dataset. We did not omit any replicates on the basis of the experimental results
Randomization	In our experiments, flies were grouped based on their genetic background or specific neuronal manipulations, and not arbitrarily assigned to treatment groups. As a result, randomization was not applicable.
Blinding	For behavioral experiments, the existing GUI was designed in a way that allowed the experimenter to recognize the genotype of individual flies, making blinding impractical. For calcium imaging and electrophysiological experiments, the experimenter was responsible for generating the necessary fly lines and performing the recordings. Different drivers, reporters, and effectors were combined by the same person conducting the experiments. Due to logistical constraints, technical challenges, and limited staffing, conducting a blinded experiment was not feasible. For anatomical experiments, data analysis was performed automatically or semi-automatically using objective quantification methods. Since the processing and quantification were not subject to experimenter bias, blinding was not necessary. For scRNA-seq data analysis, blinding was not necessary as clustering and cell type identification were performed using automated computational pipelines. Data processing, dimensionality reduction, and clustering were conducted through standardized algorithms, minimizing the potential for experimenter bias.

Reporting for specific materials, systems and methods

We require information from authors about some types of materials, experimental systems and methods used in many studies. Here, indicate whether each material, system or method listed is relevant to your study. If you are not sure if a list item applies to your research, read the appropriate section before selecting a response.

Materials & experimental systems

n/a	Involved in the study	
☐	☒	Antibodies
☒	☐	Eukaryotic cell lines
☒	☐	Palaeontology and archaeology
☐	☒	Animals and other organisms
☒	☐	Clinical data
☒	☐	Dual use research of concern

Methods

n/a	Involved in the study	
☒	☐	ChIP-seq
☒	☐	Flow cytometry
☒	☐	MRI-based neuroimaging

Antibodies

Antibodies used	Primary antibodies and dilutions used in this study:<br>-chicken anti-GFP (1:1000, Abcam #ab13970, RRID: AB_300798),<br>-rabbit anti-dsRed (1:200, Clontech #632496, RRID: AB_10013483),<br>-mouse anti-Bruchpilot (1:20, DSHB Nc82, RRID: AB_2314866),<br>-chicken anti-V5 (1:200, Fortis Life Sciences #A190-118A, RRID: AB_66741),<br>-mouse anti-V5 (1:500, Abcam #ab27671, RRID: AB_471093),<br>-rabbit anti-HA (1:200, Cell Signaling Technology #3724, RRID: AB_1549585),<br>-rabbit anti-FLAG (1:200, Abcam #ab205606, RRID: AB_2916341),<br>-rat anti-N-Cadherin (1:40, DSHB MNCD2, RRID: AB_528119),<br>-anti-GFP nanobody (1:200 for expansion microscopy, 1:500 for confocal microscopy, NanoTag Biotechnologies #N0304-At488-L, RRID: AB_2744629),<br>-rat anti-HA (1:500 for expansion microscopy, Roche 3F10, RRID: AB_2314622),<br><br>Secondary antibodies and dilutions used in this study:<br>-goat anti-chicken AF488 (1:500, Invitrogen #A11039, RRID: AB_2534096),<br>-goat anti-mouse AF488 IgG2A (1:500, Invitrogen #A21131, RRID: AB_2535771),<br>-goat anti-rabbit AF568 (1:500, Invitrogen #A11011, RRID: AB_143157),<br>-goat anti-mouse AF647 (1:500, Jackson ImmunoResearch #115-607-003, RRID: AB_2338931),<br>-goat anti-rat AF647 (1:500, Jackson ImmunoResearch #112-605-167, RRID: AB_2338404).
Validation	The following antibodies were validated in our previous study (Dombrovski et al., 2023, https://doi.org/10.1038/s41586-023-05930-y) focused on the same anatomical system (i.e., VPNs and DNs) in the same species:<br>-chicken anti-GFP (Abcam #ab13970, RRID: AB_300798),<br>-rabbit anti-dsRed (Clontech #632496, RRID: AB_10013483),<br>-mouse anti-Bruchpilot (DSHB Nc82, RRID: AB_2314866),<br>-rat anti-N-Cadherin (DSHB MNCD2, RRID: AB_528119),<br>-chicken anti-V5 (Fortis Life Sciences #A190-118A, RRID: AB_66741),

-mouse anti-V5 (Abcam #ab27671, RRID: AB_471093),

-rabbit anti-HA (Cell Signaling Technology #3724, RRID: AB_1549585),
-rat anti-HA (Roche 3F10, RRID: AB_2314622),
-goat anti-rabbit AF568 (Invitrogen #A11011, RRID: AB_143157),
-goat anti-mouse AF647 (Jackson ImmunoResearch #115-607-003, RRID: AB_2338931),

Goat anti-mouse AF488 IgG2A antibody (Invitrogen #A21131, RRID: AB_2535771) was validated for use in the Drosophila visual system neurons by Sanfilippo et al., 2024 (https://doi.org/10.1016/j.neuron.2023.12.014)

Rabbit anti-FLAG antibody (Abcam #ab205606, RRID: AB_2916341), had not been previously used in Drosophila. We validated it through MCFO experiments, confirming specific labeling of LPLC2 neurons with their well-characterized morphology and projections, ensuring its reliability in Drosophila tissues.

Anti-GFP nanobody (NanoTag Biotechnologies #N0304-At488-L, RRID: AB_2744629) was validated in tissue expansion experiments by enhancing the innate fluorescence of nuclear GFP. Rather than labeling de novo, we confirmed its specificity by matching the enhnced signal to known GFP expression patterns.

Goat anti-chicken AF488 antibody (Invitrogen #A11039, RRID: AB_2534096), was validated by staining GFP, using its innate fluorescence as a reference. This allowed us to confirm specificity by ensuring the antibody signal matched the expected GFP expression pattern.

Goat anti-rat AF647 (Jackson ImmunoResearch #112-605-167, RRID: AB_2338404) had not been previously used in Drosophila. We validated it by staining the Giant Fiber, which expressed a rat HA antibody, allowing us to confirm its specificity based on the well-characterized morphology of GF dendritic patterns.

Animals and other research organisms

Policy information about studies involving animals; ARRIVE guidelines recommended for reporting animal research, and Sex and Gender in Research

Laboratory animals	Supplementary Table 1 provides detailed descriptions of fly genotypes used in each experiment and origins of transgenic stocks, with references to specific figure panels. Details on generation of transgenic stocks can be found in the corresponding Methods section. For calcium imaging experiments, the flies recorded were between 1 to 3 days old. For electrophysiology experiments, 3-7 days old flies were used. For behavioral experiments, 3-5 days old flies were used. For scRNA-seq and FISH experiments, as well as anatomical experiments in Fig. 2i, 3d, Ext. Data Fig. 5, Ext. Data Fig. 6b, and Ext. Data Fig. 10a, animals represented a designated pupal stage (i.e., 48, 72 or 96 h APF). In all other cases, tested flies were 1-3 days old.
Wild animals	No wild animals were used in this study
Reporting on sex	For electrophysiology and calcium imaging experiments, only female flies were used. For experiments in Figure 4 and Extended Data Figure 7, only female flies were used due to genetic constraints related to transgene localization. The required transgenic constructs (UAS-Dcr2 and hsFLP for beat-VI and side-II RNAi experiments, and UAS-smHA and hsFLP for STaR experiments) are located on the X chromosome. As a result, generating males with the necessary genotype would require complex recombination strategies, making it impractical. With this exception, flies of both sexes were considered in all other experiments. None of the findings in this study are sex-specific or expected to be influenced by sex. Additionally, both connectomic datasets used in this study were obtained from female flies.
Field-collected samples	No field samples were collected for this study
Ethics oversight	No ethical approval was required because experiments were performed on Drosophila melanogaster

Note that full information on the approval of the study protocol must also be provided in the manuscript.

This checklist template is licensed under a Creative Commons Attribution 4.0 International License, which permits use, sharing, adaptation, distribution and reproduction in any medium or format, as long as you give appropriate credit to the original author(s) and the source, provide a link to the Creative Commons license, and indicate if changes were made. The images or other third party material in this article are included in the article's Creative Commons license, unless indicated otherwise in a credit line to the material. If material is not included in the article's Creative Commons license and your intended use is not permitted by statutory regulation or exceeds the permitted use, you will need to obtain permission directly from the copyright holder. To view a copy of this license, visit http://creativecommons.org/licenses/by/4.0/
