# Supplementary material for: Molecular gradients shape synaptic specificity of a visuomotor transformation
Source: Nature. 2025 Jun 4;644(8076):453–62. doi: 10.1038/s41586-025-09037-4 (PMC12350164; doi:10.1038/s41586-025-09037-4)
Supplement: Supplementary file 3 — This file provides a summary of the statistical tests conducted for each experiment, including exact P-values [file 41586_2025_9037_MOESM3_ESM.pdf]

Figure 1e, Extended Data Figure 1a
Takeoff distribution comparison at different stimulus elevations
* for p < 0.05, ** < 0.01, *** < 0.001 and **** < 0.0001.

Azimuth 0°, percentage of short-mode takeoffs comparison
Chi^2 result of the contingency table: 31.95677140967898, p-value: 1.952412835258972e-06
The p-value (Bonf.) indicates the application of the post-hoc Bonferroni correction for multiple comparisons.

Comparison (elevation,°)	p-value	p-value (Bonf.)	Significance marker
-30, 0	0.954995	1.000000	NS
-30, 23	0.369536	1.000000	NS
-30, 45	0.133405	1.000000	NS
-30, 77	0.000088	0.000879	***
0, 23	0.311570	1.000000	NS
0, 45	0.024924	0.249237	NS
0, 77	0.000000	0.000003	****
23, 45	0.728703	1.000000	NS
23, 77	0.000568	0.005679	**
45, 77	0.000004	0.000037	****

Azimuth 90°, percentage of short-mode takeoffs comparison
Chi^2 result of the contingency table: 33.758764514460694, p-value: 8.350835665073025e-07
The p-value (Bonf.) indicates the application of the post-hoc Bonferroni correction for multiple comparisons.

Comparison (elevation,°)	p-value	p-value (Bonf.)	Significance marker
-30, 0	0.367050	1.000000	NS
-30, 23	0.515520	1.000000	NS
-30, 45	0.323886	1.000000	NS
-30, 77	0.000660	0.006604	**
0, 23	0.030524	0.305237	NS
0, 45	0.000323	0.003225	**
0, 77	0.000000	0.000000	****
23, 45	0.953205	1.000000	NS
23, 77	0.002148	0.021484	*
45, 77	0.000040	0.000399	***

Extended Data Figure 1c
* for p < 0.05, ** < 0.01, *** < 0.001 and **** < 0.0001.

Azimuth 0°, percentage of total takeoff % takeoffs comparison
Chi2 result of the contingency table: 31.95677140967898, p-value: 1.952412835258972e-06
The p-value (Bonf.) indicates the application of the post-hoc Bonferroni correction for multiple comparisons.

Comparison (elevation,°)	p-value	p-value (Bonf.)	Significance marker
-30, 0	0.170595	1.000000	NS
-30, 23	0.000028	0.000283	***
-30, 45	0.000007	0.000072	****
-30, 77	0.000025	0.000246	***
0, 23	0.000272	0.002720	**

0, 45	0.000006	0.000064	****
0, 77	0.000253	0.002531	**
23, 45	0.233901	1.000000	NS
23, 77	0.802820	1.000000	NS
45, 77	0.146043	1.000000	NS

Azimuth 90°, percentage of total takeoff % takeoffs comparison
Chi2 result of the contingency table: 31.95677140967898, p-value: 1.952412835258972e-06
The p-value (Bonf.) indicates the application of the post-hoc Bonferroni correction for multiple comparisons.

Comparison (elevation,°)	p-value	p-value (Bonf.)	Significance marker
-30, 0	0.739598	1.000000	NS
-30, 23	0.000000	0.000000	****
-30, 45	0.000003	0.000026	****
-30, 77	0.065939	0.659390	NS
0, 23	0.000000	0.000000	****
0, 45	0.000000	0.000000	****
0, 77	0.061991	0.619908	NS
23, 45	0.001984	0.019835	*
23, 77	0.000387	0.003870	**
45, 77	0.062691	0.626907	NS

Figure 1f, Extended Data Figure 1e
Giant Fiber response to looming stimuli at different stimulus elevations
* for p < 0.05, ** < 0.01, *** < 0.001 and **** < 0.0001.

Comparison of peak responses:
Repeated-measures one-way ANOVA, p=0.004756, significance marker: **
The p-value (Sidak) indicates the application of the post-hoc Sidak correction for multiple comparisons.

Comparison (elevation,°)	p-value	p-value (Sidak)	Significance marker
-25, 0	0.064726	0.181880	NS
-25, 25	0.013007	0.038515	*
0,25	0.061421	0.173176	NS

Comparison of areas under curve (mV x ms):
Repeated-measures one-way ANOVA, p= 0.010014, significance marker: *
The p-value (Sidak) indicates the application of the post-hoc Sidak correction for multiple comparisons.

Comparison (elevation,°)	p-value	p-value (Sidak)	Significance marker
-25, 0	0.075706	0.210357	NS
-25, 25	0.016800	0.049558	NS
0,25	0.151799	0.389767	NS

Extended Data Figure 1f-h
Comparison of escape takeoff behaviors in LPLC2-silenced flies
* for p < 0.05, ** < 0.01, *** < 0.001 and **** < 0.0001.

Takeoff distribution comparison
Mann-Whitney U test
Empty Gal4>UAS-TNT vs LPLC2 Gal4>UAS-TNT

Comparison (elevation, °)	Mann-Whitney U statistic	p-value	Significance marker
0	1649.0	0.000272	***
45	2231.5	0.011792	*
77	2320.0	0.004047	**

Short-mode takeoff percentage comparison
The p-value (Bonf.) indicates the application of the post-hoc Bonferroni correction for multiple comparisons.
Empty Gal4>UAS-TNT
Chi2 result of the contingency table: 4.651964208254206,, p-value: 0.09768745766941091

Comparison (elevation,°)	p-value	p-value (Bonf.)	Significance marker
0, 45	0.389335	1.000000	NS
0, 77	0.049921	0.149762	NS
45, 77	0.320999	0.962998	NS

LPLC2 Gal4>UAS-TNT
Chi2 result of the contingency table: 0.6969626396197942, p-value: 0.7057590986149207

Comparison (elevation,°)	p-value	p-value (Bonf.)	Significance marker
0, 45	0.584055	1.000000	NS
0, 77	0.950082	1.000000	NS
45, 77	0.807008	1.000000	NS

Total takeoff percentage comparison
Empty Gal4>UAS-TNT vs LPLC2 Gal4>UAS-TNT

elevation,°	Chi2 value	p-value	Significance marker
0	27.267213938266572	1.771906e-07	****
45	67.95681647353312	1.671157e-16	****
77	42.19240434447577	8.272068e-11	****

Figure 2f-h, Extended Data Figure 4b-c
FISH data, 12 gene pairs at 3 developmental time points. Correlation between gene expression assessed using a standard linear mixed-effects model (LMM) and generalized linear mixed model (GLMM, to account for data overdispersion) with response variable following a negative binomial distribution, both models accounting for random effects by brain.
Statistically nonsignificant results are shown in **bold** (p>0.05). Negative values for t-statistics and z-statistics indicate a negative correlation between the gene pairs at the specified developmental time points.

Gene Pair, Time	LMM t-statistic	LMM p-value	GLMM z-statistic	GLMM p-value
beat-VI/dpr13, 48h APF	15.678	6.15e-49	5.371	9.99e-09
beat-VI/dpr13, 72h APF	10.291	5.99e-23	8.121	4.64e-16
beat-VI/dpr13, 96h APF	7.619	1.61e-13	6.529	6.64e-11
fz2/beat-VI, 48h APF	14.960	1.81e-42	11.224	3.12e-29
fz2/beat-VI, 72h APF	8.074	1.34e-14	4.748	2.05e-06

fz2/beat-VI, 96h APF	3.965	8.86e-05	3.495	0.000473
tei/dpr13, 48h APF	15.311	7.84e-40	8.540	1.34e-17
tei/dpr13, 72h APF	6.818	6.07e-11	4.209	2.57e-05
tei/dpr13, 96h APF	11.423	3.60e-27	7.205	5.82e-13
nAChRalpha5/beat-VI, 48h APF	12.543	3.87e-30	6.853	7.21e-12
nAChRalpha5/beat-VI, 72h APF	15.012	1.71e-39	7.210	5.59e-13
nAChRalpha5/beat-VI, 96h APF	9.360	9.65e-19	8.019	1.07e-15
dpr17/dpr13, 48h APF	-4.536	7.77e-06	-4.590	4.43e-06
dpr17/dpr13, 72h APF	-4.411	1.37e-05	-5.081	3.75e-07
dpr17/dpr13, 96h APF	-7.527	3.08e-13	-5.197	2.03e-07
beat-VI/dpr17, 48h APF	-5.260	2.25e-07	-5.789	7.11e-09
beat-VI/dpr17, 72h APF	-3.036	0.002588	-3.069	0.002145
beat-VI/dpr17, 96h APF	-0.663	**0.507632**	-0.501	**0.616698**
SiaT/dpr13, 48h APF	-9.572	7.29e-20	-3.760	0.000171
SiaT/dpr13, 72h APF	-4.410	1.37e-05	-5.081	3.75e-05
SiaT/dpr13, 96h APF	-7.519	3.27e-13	-3.742	0.000182
beat-VI/SiaT, 48h APF	-5.428	8.74e-08	-5.085	3.68e-07
beat-VI/SiaT, 72h APF	-3.325	0.000968	-3.365	0.000765
beat-VI/SiaT, 96h APF	-3.573	0.000403	-3.307	0.000941
Cad87A/SiaT, 48h APF	-4.466	9.74e-06	-5.170	2.35e-07
CG30419/beat-VI, 48h APF	-8.421	4.51e-14	-7.036	1.97e-12
CG30419/beat-VI, 72h APF	-2.863	0.004402	-2.914	0.003571
CG30419/beat-VI, 96h APF	-4.718	3.04e-06	-3.808	0.000139
dpr13/CG30419, 48h APF	-6.818	5.02e-11	-4.697	2.64e-06
dpr13/CG30419, 72h APF	-6.045	3.89e-09	-6.385	1.72e-10
dpr13/CG30419, 96h APF	-3.122	0.001957	-1.826	**0.067814**
mbl/dpr13, 48h APF	2.507	0.012457	1.647	**0.099568**
mbl/dpr13, 72h APF	0.687	**0.492210**	0.236	**0.813567**
mbl/dpr13, 96h APF	0.849	**0.396589**	0.696	**0.486700**

Extended Data Figure 4e-h.
FISH puncta counts in sparsely labeled dorsal and ventral LPLC2 neurons. Unpaired t-test with Welch's correction.

e-f

Comparison	p-value	Significance marker
Dpr13 dorsal vs Dpr13 ventral	0.002233746477676	**
Beat-VI dorsal vs Beat-VI ventral	0.000232408142355	***

Extended Data Figure 4j-l.
Sparsely labeled LPLC2 co-localized with Beat-VI and Dpr13 protein traps. Unpaired t-test with Welch's correction.

Comparison	p-value	Significance marker
Dpr13 dorsal vs Dpr13 ventral	0.003511429510734	**
Beat-VI dorsal vs Beat-VI ventral	0.000221814011944	***

Figure 3e-g, m, o
* for p < 0.05, ** < 0.01, *** < 0.001 and **** < 0.0001
Comparison of LPLC2-GF axo-dendritic overlap volume
e. Unpaired t-test with Welch's correction.

Comparison	p-value	Significance marker	Difference between means ± 95% CI
DIP-$\varepsilon^{-/-}$ 48h vs DIP-ε+/+ 48h	0.000323566988959	***	-74.678 ± 33.775
DIP-$\varepsilon^{-/-}$ 48h vs DIP-ε+/+ 72h	2.1380632369e-5	****	-122.306 ± 38.003
DIP-$\varepsilon^{-/-}$ 48h vs DIP-ε+/+ 96h	3.3436e-11	****	-178.951 ± 25.807

f. Unpaired t-test with Welch's correction.

Comparison	p-value	Significance marker
Control KK vs *DIP-ε* RNAi KK	1.346917e-09	****
Control GD vs *DIP-ε* RNAi GD	7.58741305e-07	****

g. One-way ANOVA followed by Tukey's (HSD) test for post-hoc pairwise comparisons.
F=63.7536190559813, p=3.64e-13

Comparison	p-value	Significance marker
DIP-$\varepsilon^{-/-}$+ UAS-DIP-ε vs. DIP-$\varepsilon^{-/-}$+ GF-GAL4	0.587797888152264	NS
DIP-$\varepsilon^{-/-}$+ UAS-DIP-ε vs. DIP-$\varepsilon^{-/-}$+ GF-GAL4 + UAS-DIP-ε	3.76e-12	****
DIP-$\varepsilon^{-/-}$+ GF-GAL4 vs. DIP-$\varepsilon^{-/-}$+ GF-GAL4 + UAS-DIP-ε	2.1884e-11	****

m, o. Unpaired t-test with Welch's correction.

Comparison	p-value	Significance marker
LPLC2>Control vs. LPLC2>UAS-*dpr13*	0.022640	*
LPLC2>Control vs. LPLC2>UAS-*DIP-ε*	0.000503	***

Extended Data Figure 6
* for p < 0.05, ** < 0.01, *** < 0.001 and **** < 0.0001.
g. Comparison of LPLC2-GF axo-dendritic overlap volume in controls vs *dpr13^{null}* animals
Unpaired t-test with Welch's correction.

Comparison	p-value	Significance marker	Difference between means ± 95% CI
Control 96h vs *dpr13^{null}* 96h	0.4045	NS	16.34±19.27

l. HCR-FISH puncta in LPLC2>Control vs LPLC2>UAS-*DIP-ε*
Unpaired t-test with Welch's correction.

Comparison	p-value	Significance marker	Difference between means ± 95% CI
UAS-control vs UAS-*DIP-ε*	0.00012142240138	***	4.49 ± 0.384

Extended Data Figure 7.
* for p < 0.05, ** < 0.01, *** < 0.001 and **** < 0.0001.
Sparse STaR in LPLC2 in control and dpr13 null background
Unpaired t-test with Welch's correction.

Comparison	p-value	Significance marker
Fraction of T-bars overlapping with the GF, control vs *dpr13*^{null}	0.239028977465117	NS
Number of T-bars overlapping with the GF per LPLC2 neuron, control vs *dpr13*^{null}	0.371744277321679	NS
Total number of T-bars per LPLC2 neuron, control vs *dpr13*^{null}	0.785108760643073	NS

Sparse STaR in LPLC2 in GF>control and GF>DIP-ε RNAi
Unpaired t-test with Welch's correction.

Comparison	p-value	Significance marker
Fraction of T-bars overlapping with the GF, UAS-control RNAi vs UAS-*DIP-ε* RNAi	0.000047330688065	****
Number of T-bars overlapping with the GF per LPLC2 neuron, UAS-control RNAi vs UAS-*DIP-ε* RNAi	0.00002982838174	****
Total number of T-bars per LPLC2 neuron, UAS-control RNAi vs UAS-*DIP-ε* RNAi	0.12458367222565	NS

Figure 3h, i
* for p < 0.05, ** < 0.01, *** < 0.001 and **** < 0.0001.
h. Comparison of peak amplitude of GF in controls vs *DIP-ε*^{null} animals
Mann-Whitney U test

Comparison (in r/v = 40 ms)	Mann-Whitney U statistic	p-value	Significance marker
Control vs *DIP-ε*^{null}	4	0.0303	*

i. Comparison of peak amplitude of GF in controls vs DIP-ε-RNAi animals
Mann-Whitney U test

Comparison (in r/v = 40 ms)	Mann-Whitney U statistic	p-value	Significance marker
Control vs *DIP-ε*-RNAi	2	0.03175	*

Extended Data Figure 8 - b, d
* for p < 0.05, ** < 0.01, *** < 0.001 and **** < 0.0001.
b. Comparison of peak amplitude of GF in controls vs *DIP-ε*^{null} animals
Mann-Whitney U test

Comparison (in each r/v, ms)	Mann-Whitney U statistic	p-value	Significance marker
10	8	0.149	NS
20	6	0.07323	NS
40	4	0.0303	*
80	14	0.6389	NS

b. Comparison of peak amplitude of GF in controls vs DIP-ε-RNAi animals
Mann-Whitney U test

Comparison (in each r/v, ms)	Mann-Whitney U statistic	p-value	Significance marker
10	6	0.2222	NS
20	1	0.01587	*
40	2	0.03175	*
80	2	0.03175	*

Figure 3j-l, n
Takeoff distribution comparison
* for p < 0.05, ** < 0.01, *** < 0.001 and **** < 0.0001.
Mann-Whitney U test
DL vs *DIP-ε*^{null}

Comparison (elevation, °)	Mann-Whitney U statistic	p-value	Significance marker
0	1102.5	2.762732e-07	****
45	1084.5	8.165668e-09	****
77	1588.0	1.977863e-04	***

Empty Gal4>UAS-DIP-ε-RNAi vs GF Gal4> UAS-*DIP-ε*-RNAi

Comparison (elevation, °)	Mann-Whitney U statistic	p-value	Significance marker
0	3294.0	0.031027	*
45	3259.5	0.334079	NS
77	2902.0	1.920117e-04	***

Empty Gal4>UAS-Dpr13 vs LPLC2 Gal4>UAS-*dpr13*

Comparison (elevation, °)	Mann-Whitney U statistic	p-value	Significance marker
0	3604.0	4.625898e-04	***
45	3613.0	3.834724e-02	*
77	3181.5	0.941107	NS

DL vs *dpr13*^{null}

Comparison (elevation, °)	Mann-Whitney U statistic	p-value	Significance marker
0	4095.0	0.005558	**
45	2302.5	0.426707	NS
77	2110.5	0.771449	NS

Short-mode takeoff percentage comparison
* for p < 0.05, ** < 0.01, *** < 0.001 and **** < 0.0001.
The p-value (Bonf.) indicates the application of the post-hoc Bonferroni correction for multiple comparisons.
DL

Chi2 result of the contingency table: 8.684853691788211, p-value: 0.01300492891730162

Comparison (elevation,°)	p-value	p-value (Bonf.)	Significance marker
0, 45	0.226045	0.678134	NS
0, 77	0.007282	0.021846	*
45, 77	0.211155	0.633466	NS

DIP-ε^{null} Chi2 result of the contingency table: 9.80602901959989, p-value: 0.007424169073861333

Comparison (elevation,°)	p-value	p-value (Bonf.)	Significance marker
0, 45	1.000000	1.000000	NS
0, 77	0.038308	0.114924	NS
45, 77	0.082882	0.248647	NS

Empty Gal4>UAS-*DIP-ε*-RNAi
Chi2 result of the contingency table: 6.775081042471815, p-value: 0.03379168471070383

Comparison (elevation,°)	p-value	p-value (Bonf.)	Significance marker
0, 45	0.367950	1.000000	NS
0, 77	0.015671	0.047012	*
45, 77	0.188825	0.566475	NS

GF Gal4> UAS-*DIP-ε*-RNAi
Chi2 result of the contingency table: 0.1740084285332571, p-value: 0.9166732325195991

Comparison (elevation,°)	p-value	p-value (Bonf.)	Significance marker
0, 45	1.000000	1.000000	NS
0, 77	0.873503	1.000000	NS
45, 77	0.902151	1.000000	NS

Empty Gal4>UAS-*dpr13*
Chi2 result of the contingency table: 13.237938025041398, p-value: 0.00133480640514174

Comparison (elevation,°)	p-value	p-value (Bonf.)	Significance marker
0, 45	0.164585	0.493755	NS
0, 77	0.000583	0.001750	**
45, 77	0.064840	0.194521	NS

LPLC2 Gal4>UAS-*dpr13*
Chi2 result of the contingency table: 0.28989715660728177, p-value: 0.8650667751681032

Comparison (elevation,°)	p-value	p-value (Bonf.)	Significance marker
0, 45	0.770994	1.000000	NS
0, 77	1.000000	1.000000	NS
45, 77	0.752068	1.000000	NS

DL
Chi2 result of the contingency table: 7.571740071036096, p-value: 0.022689

Comparison (elevation,°)	p-value	p-value (Bonf.)	Significance marker
0, 45	0.186470	0.559409	NS
0, 77	0.011414	0.034243	*
45, 77	0.328558	0.985674	NS

dpr13^{null}

Chi2 result of the contingency table: 0.9491983211739197, p-value: 0.622134

Comparison (elevation,°)	p-value	p-value (Bonf.)	Significance marker
0, 45	1.000000	1.000000	NS
0, 77	0.456835	1.000000	NS
45, 77	0.565391	1.000000	NS

Extended Data Figure 9

Total takeoff percentage comparison

* for p < 0.05, ** < 0.01, *** < 0.001 and **** < 0.0001.

DL vs *DIP-ε*^{null}

elevation,°	Chi2 value	p-value	Significance marker
0	2.464751	0.116426	NS
45	9.476042	0.002082	**
77	23.707169	1.121639e-06	****

Empty Gal4>UAS-DIP-ε-RNAi vs *GF Gal4> UAS-DIP-ε-RNAi*

elevation,°	Chi2 value	p-value	Significance marker
0	2.200417	0.137973	NS
45	0.002766	0.958060	NS
77	5.731744	0.016661	*

Empty Gal4>UAS-dpr13 vs *LPLC2 Gal4>UAS-dpr13*

elevation,°	Chi2 value	p-value	Significance marker
0	15.674529	7.523051e-05	****
45	10.933662	9.443267e-04	***
77	5.1404340	0.023375	*

DL vs *dpr13*^{null}

elevation,°	Chi2 value	p-value	Significance marker
0	11.988401	5.353269e-04	***
45	1.405422	0.235818	NS
77	0.009356	0.922942	NS

Figure 4d

* for p < 0.05, ** < 0.01, *** < 0.001 and **** < 0.0001.

Comparison of Lop4 dendritic branch length between controls and *beat-VI* RNAi

Unpaired t-test with Welch's correction.

Comparison	p-value	Significance marker	Difference between means ± 95% CI
Control vs *beat-VI* RNAi Dorsal	2.95239e-8	****	−13.4955±2.579
Control vs *beat-VI* RNAi Central	2.263677e-8	****	−9.5170±1.376
Control vs *beat-VI* RNAi Ventral	0.7886	NS	−0.2033±1.089

Figure 5e

Comparison of DSI in dorsal (16) and ventral (40) positions (Position) in controls, *beat-VI* RNAi, and UAS-*beat-VI* (Genotype).

Two-way repeated-measures ANOVA (rANOVA) followed by unpaired t-test adjusted with Bonferroni method for post-hoc comparisons. Position: $Chi^2 = 4.6031$, p = 0.1001; Genotype: $Chi^2 = 3.2581$, p = 0.1961; Position*Genotype: $Chi^2 = 9.8026$, p = 0.0074.

Comparison	p-value (Bonf.)	Significance marker	Difference between means ± SE	Cohen's *d*
Control Dorsal vs Control Ventral	0.02	*	-0.5720±0.173	-1.7095
beat-VI RNAi Dorsal vs *beat-VI* RNAi Ventral	1.0000	NS	0.0785±0.152	0.2345
UAS-*beat-VI* Dorsal vs UAS-*beat-VI* Ventral	1.0000	NS	-0.0175±0.098	-0.0523
Control Dorsal vs UAS-*beat-VI* Dorsal	1.0000	NS	-0.0867±0.166	-0.2591
Control Dorsal vs UAS-*beat-VI* Ventral	1.0000	NS	-0.1042±0.166	-0.3114
Control Dorsal vs *beat-VI* RNAi Dorsal	0.8355	NS	-0.3527±0.179	-1.0541
Control Dorsal vs *beat-VI* RNAi Ventral	1.0000	NS	-0.2743±0.202	-0.8196
Control Ventral vs UAS-*beat-VI* Dorsal	0.2351	NS	0.4853±0.195	1.4504
Control Ventral vs UAS-*beat-VI* Ventral	0.2945	NS	0.4678±0.195	1.3981
Control Ventral vs *beat-VI* RNAi Dorsal	1.0000	NS	0.2193±0.206	0.6555
Control Ventral vs *beat-VI* RNAi Ventral	1.0000	NS	0.2978±0.226	0.8899
UAS-*beat-VI* Dorsal vs *beat-VI* RNAi Dorsal	1.0000	NS	-0.2660±0.154	-0.7950
UAS-*beat-VI* Dorsal vs *beat-VI* RNAi Ventral	1.0000	NS	-0.1876±0.180	-0.5605
UAS-*beat-VI* Ventral vs *beat-VI* RNAi Dorsal	1.0000	NS	-0.2485±0.154	-0.7426
UAS-*beat-VI* Ventral vs *beat-VI* RNAi Ventral	1.0000	NS.	-0.1700±0.180	-0.5082

Pairwise t-test adjusted with Bonferroni method for post-hoc comparisons on the difference between ventral (40) and dorsal (16) positions.
UAS-*beat-VI* vs *beat-VI* RNAi

Comparison	estimate	SE	df	t-ratio	p-value (Bonf.)	Significance marker
UAS-*beat-VI* vs *beat-VI* RNAi	-0.096	0.181	85.3	-0.531	1.0000	NS
Control vs *beat-VI* RNAi	-0.650	0.230	90.1	-2.828	0.0173	*
Control vs UAS-*beat-VI*	-0.555	0.198	87.7	-2.794	0.0192	*

Figure 5h
Comparison of peak responses to looming stimuli above (dorsal) and below (ventral) the fly eye's equator (Position) in controls, *beat-VI* RNAi, and UAS-*beat-VI* (Genotype).
Two-way repeated-measures ANOVA (rANOVA) followed by unpaired t-test adjusted with Bonferroni method for post-hoc comparisons. Position: $Chi^2 = 75.7508$, $p < 0.00001$; Genotype: $Chi^2 = 2.9296$, p = 0.2311; Position*Genotype: $Chi^2 = 0.3302$, p = 0.8478.

Comparison	p-value	Significance marker	Difference between means ± SE

Control Dorsal vs Control Ventral	1.0000	NS	0.0166±0.013
beat-VI RNAi Dorsal vs *beat-VI* RNAi Ventral	1.0000	NS	0.0074±0.012
UAS-*beat-VI* Dorsal vs UAS-*beat-VI* Ventral	1.0000	NS	0.0147±0.008

Extended Data Figure 10b-c

* for p < 0.05, ** < 0.01, *** < 0.001 and **** < 0.0001.

b. Comparison of LPLC2-GF axo-dendritic overlap volume in controls vs *side-II*^{null} animals
Unpaired t-test with Welch's correction.

Comparison	p-value	Significance marker	Difference between means ± 95% CI
side-II^{null} vs control	0.3884	NS	12.47±28.40

c. Comparison of LPLC2-GF axo-dendritic overlap volume in controls vs *beat-VI* RNAi in LPLC2
One-way ANOVA followed by Tukey's (HSD) test for post-hoc pairwise comparisons. F= 1.036, p=0.3657. * for p < 0.05, ** < 0.01, *** < 0.001 and **** < 0.0001.

Comparison	p-value	Significance marker
UAS-Control (KK) vs. *beat-VI* RNAi (KK)	0.1490	NS
UAS-Control (GD) vs. *beat-VI* RNAi (GD)	0.2956	NS

Extended Data Figure 10g.

* for p < 0.05, ** < 0.01, *** < 0.001 and **** < 0.0001.

Comparison of Lop4 dendritic branch length between UAS-controls and UAS-*beat-VI* in LPLC2
Unpaired t-test with Welch's correction.

Comparison	p-value	Significance marker
dorsal UAS-control vs dorsal UAS-*beat-VI*	0.073174108727134	NS
central UAS-control vs central UAS-*beat-VI*	0.642797831746797	NS
ventral UAS-control vs ventral UAS-*beat-VI*	0.006134577342864	**
